# Sex differences in cancer incidence and survival: a Danish nationwide population-based study assessing 35 cancer sites

**DOI:** 10.1038/s41416-026-03429-7

**Published:** 2026-04-27

**Authors:** Fie Stegenborg, Pernille Envold Bidstrup, Klaus Rostgaard, Òlafur Birgir Davidsson, Carsten Utoft Niemann, Ismail Gögenur, Erik Jakobsen, Susanne Oksbjerg Dalton, Henrik Hjalgrim

**Affiliations:** 1 Cancer Survivorship, Danish Cancer Institute, Danish Cancer Society, Copenhagen, Denmark; 2 Department of Public Health, University of Copenhagen, Copenhagen, Denmark; 3 Psychological Aspects of Cancer, Cancer Survivorship, Danish Cancer Institute, Danish Cancer Society, Copenhagen, Denmark; 4 Hematology and Childhood Cancer, Danish Cancer Institute, Danish Cancer Society, Copenhagen, Denmark; 5 Department of Hematology, Copenhagen University Hospital, Rigshospitalet, Copenhagen, Denmark; 6 Department of Clinical Medicine, University of Copenhagen, Copenhagen, Denmark; 7 Department of Surgery, Center for Surgical Science, Zealand University Hospital, Koege, Denmark; 8 Department of Thoracic Surgery, Odense University Hospital, Odense, Denmark; 9 Danish Research Center for Equality in Cancer, Department of Clinical Oncology and Palliative Care, Zealand University Hospital, Naestved, Denmark

**Keywords:** Risk factors, Epidemiology, Cancer epidemiology

## Abstract

**Background:**

Sex differences in cancer incidence and survival have been documented, but underlying mechanisms remain unclear. We examined sex differences in incidence and survival for non-sex-specific cancers and the role of socioeconomic factors and comorbidity.

**Methods:**

All individuals living in Denmark from 2004–2020 were included. Incidence rate ratios (IRRs) and excess mortality ratios (EMRs) for 35 cancers were estimated using Poisson regression adjusted for age and year. Modification of associations between sex and death by cohabitation, education and comorbidity was assessed.

**Results:**

A total of 7,339,667 individuals were followed for 99,832,998 person-years, during which 355,339 were registered with a primary malignancy (197,375 males, 157,964 females). Males had a 52% higher risk of cancer and upon cancer diagnosis 10% higher mortality than females. IRRs were elevated (with confidence intervals excluding the null) for 24 cancers and EMRs for 16 cancers in males. One in six cancers and one in five cancer deaths in males could have been avoided if male rates matched female rates. Disparities were greatest among males living alone, particularly for alcohol- and smoking-related cancers.

**Conclusion:**

Sex differences in cancer must be addressed and translated into interventions that promote equality between sexes, specifically focusing on socioeconomically vulnerable males.

## Introduction

Despite increasing evidence that an individual’s sex is one of the most important determinants of disease risk and survival across most fields of medicine, the independent significance of biological sex is rarely considered in epidemiological or clinical research [1]. This is true also in the context of cancer, where the European Society of Medical Oncology (ESMO) recently called for investigations into the causes and consequences of sex-specific differences in cancer incidence and survival [2].

While much needed attention to the many forms of discrimination that may marginalise women with cancer [3] has been called for over the last decade, men at the same time may also encounter specific and similarly underexplored disadvantages. Indeed, several studies have shown that in populations with equal access to advanced healthcare, males are at higher risk of both developing a wide range of cancers and of succumbing to them compared with females [4–9]. In this regard, it has been demonstrated that males also face a higher risk of developing a second primary cancer than do females [10].

The mechanisms underlying sex-specific differences in cancer risk and survival remain poorly understood but likely involve a combination of mostly immutable biological factors related to sex and various modifiable socioeconomic factors related to gender [6, 8]. These characteristics and exposures likely affect the entire cancer trajectory: from the prevalence and consequences of exposure to cancer risk factors to participation in cancer screening [11] and other health-seeking behaviours as well as delivery of and adherence to cancer treatment [12]. In this context, socioeconomic and health characteristics such as cohabitation status, education level and comorbidity are critical for disease incidence and outcomes in general [13, 14]. While this is also the case for numerous types of cancers in both sexes [13], the extent to which socioeconomic and non-cancer health disparities differ between males and females and contributes to the observed male cancer disadvantage has not previously been reported.

In a population-based nationwide study, we therefore estimated sex differences in cancer incidence and survival across 35 non-sex-specific cancer sites. We further explored how any such differences varied by cohabitation status, education level and comorbidity for males and females.

## Methods

### Study population and data sources

Since 1968, the Danish Civil Registration System (CRS) has included information on vital status and addresses of all individuals living in Denmark by means of unique Personal Identification Numbers (PINs) assigned at birth to all residents. The PIN is also used by other nationwide health and social registers allowing these to be linked at the level of the individual [15]. Through the CRS, we identified all individuals living in Denmark at some time from 2004 to 2020.

Information on cancer diagnoses was obtained through the nationwide Danish Cancer Register [16], which holds information on all identified cancers in Denmark since 1943. We excluded all individuals diagnosed with cancer (other than basal cell skin carcinoma) before 2004. Next, we followed the cohort and obtained information on all first primary cancers (incl. multiple primary cancers on the same date) in individuals aged 0–99 years, diagnosed between 2004 and 2020 (Supplementary Fig. 1). We considered 35 non-sex-specific cancer types categorised according to the International Classification of Diseases, Tenth Revision (ICD-10) [17]. Leukaemias were categorised based on the International Classification of Diseases for Oncology Third Edition (ICD-O-3) [18] (Supplementary Table 1). To investigate sex differences in cancers based on aetiological risk factors that were classified as either group 1 (carcinogenic to humans) or group 2A (probably carcinogenic to humans) by the International Agency for Research on Cancer (IARC) [19], we categorised relevant cancers into four aetiological groups: alcohol, smoking, diet, or viruses (Supplementary Table 2).

From the CRS [15], we obtained information on dates of birth, immigration, emigration, cohabitation status, death and sex [15]. While individuals in Denmark can legally change gender, the current system only provides the possibility to categorise as either male or female. Thus, in the present study, sex refers to the legal sex of the individual registered in the CRS. Information on education level and comorbidity were obtained from the Danish Education Register administered by Statistics Denmark [20] and the Danish National Patient Register [21], respectively. Cohabitation status was updated on 31 December each year during the study period to allow for changes in individuals’ living arrangements over time. For individuals who were diagnosed with cancer, the last recorded cohabitation status used in the analyses was the status registered on 31 December of the preceding year. We categorised cohabitation status as living alone or living with a partner (defined as married (both opposite and same-sex), registered partnership (same-sex), or cohabiting individuals sharing a child, or two people of opposite sex not closely related with <15 years age difference). Education level was obtained only for individuals with cancer, and was measured the year before diagnosis, to ensure that early cancer symptoms did not affect the education level of the individual, categorised as short (mandatory school education for up to 7 or 9 years for people born before or after 1 January 1958, respectively), medium (senior high school or vocational education, 8–12 years), or long (higher education, >12 years). Similarly, comorbidity was obtained only for individuals with cancer and was measured by the Charlson’s Comorbidity Index (CCI) based on hospital contacts from 5 years to 3 months before cancer diagnosis and grouped according to the CCI score into 0, 1 and 2+ [22]. In analyses where the effect of cohabitation status, education level and comorbidity were assessed, the study population was restricted to individuals with cancer aged ≥30 years at diagnosis and born after 1920 due to incomplete information on education for earlier birth cohorts. We further excluded individuals with missing information on cohabitation status or education, as well as individuals who immigrated less than 5 years prior to diagnosis to ensure identification of potential comorbidities 5 years before diagnosis (Supplementary Table 3).

### Statistical analyses

#### Cancer incidence

We followed the study cohort from January 1, 2004, date of birth, or immigration, whichever occurred last, until date of the first primary cancer diagnosis, emigration, age 100 years, death or end of study period on December 31, 2020, whichever occurred first. From this, we estimated incidence rates (IRs) separately for males and females per 100,000 person-years, stratified by age in 1-year intervals, calendar year and cohabitation status (the latter only for individuals aged $\ge$ 30 years), which were then age-standardised to the Danish population in 2010 [23]. We assessed sex-specific differences in cancer incidence on both the multiplicative and the additive scale. Male-to-female incidence rate ratios (IRR_M:F_) were estimated using Poisson regression, adjusted for age at diagnosis and year of diagnosis. IR-differences (IRD_M:F_) were defined as the differences in age-standardised incidence rates between males and females per 100,000 person-years in the population. Lifetime cancer risk before the age of 100 years was calculated, taking into account population-based mortality rates and the number of potentially avoidable cases in males, had they shared the same age-specific cancer incidence rates as females, was estimated.

#### Cancer survival

We followed all individuals in the cohort diagnosed with a first primary cancer (or multiple primaries at the same date) from the date of diagnosis to date of death, emigration, or end of follow-up (5 years after diagnosis or December 31, 2020), whichever came first. Relative survival (RS) was calculated as the ratio of observed survival among individuals diagnosed with cancer to the expected survival in the general population. The expected survival was derived using the Ederer II method [24], stratified by sex, age in 1-year intervals, calendar year and cohabitation status (the latter only for individuals aged $\ge$ 30 years). RS-differences (RSD_M-F_) were estimated as the differences in age-standardised relative survival rates between males and females. Sex differences in cancer mortality were estimated as excess mortality ratios (EMR_M:F_) with follow-up up to 5 years after diagnosis using Poisson regression, comparing males to females. EMRs was defined as the hazard of additional deaths due to cancer diagnosis [24]. We estimated EMRs for all ages combined (0–99 years) and by age groups, using both 50 years (18–49 and 50–99) and 65 years (18–64 years and 65–99) as cut points. Due to few observations, we were unable to estimate RS_M:F_ and EMR_M:F_ for ages 0–17 years. We calculated the lifetime risk of dying from cancer before the age of 100 years, accounting for the mortality rates in the population. In a counterfactual analysis, we estimated the potential number of males that could have survived if they had the same excess death rates as females.

#### Aetiological group, cohabitation, education and comorbidity

The relationship between IRR_M:F_ and EMR_M:F_ was visualised by plotting the estimates of each cancer according to their aetiological risk factor(s) and cohabitation status. We assessed potential effect modifications of sex on relative risks (RRs) of death up to 5 years after diagnosis according to cohabitation status, education level and comorbidity, respectively, on both the multiplicative scale (by means of RRs) and the additive scale (by means of relative excess risks due to interaction [RERIs]) [25].

For all estimates, we reported 95% confidence intervals (CIs). All statistical analyses were conducted in R version 4.3.2 (R Core Team. 2023. Vienna, Austria).

## Results

We followed 7,339,667 individuals for a total of 99,832,998 person-years, during which 355,339 individuals were registered with a first primary cancer (197,375 males and 157,964 females) before the age of 100 years. Some individuals had more than one first primary cancer registered at the same date, thus we identified 197,797 cancers in males and 158,183 cancers in females (Supplementary Fig. 1). The most common cancers in males were lung and trachea (*n* = 31,256), colon (*n* = 21,515) and non-melanoma skin cancer (*n* = 18,252), and the most common cancers in females were lung and trachea (*n* = 30,141), colon (*n* = 21,534) and malignant melanoma (*n* = 16,903). For all examined cancers, the mean age at diagnosis was 67 years for males (Standard deviation (SD) 14) and 66 years for females (SD 16) (Table 1 and Supplementary Table 4).

**Table 1:** Characteristics of Danish males and females diagnosed with a non-sex-specific cancer site in 2004–2020.

		Male	Female
		*n* (%)^a^	*n* (%)^a^
Age group	0–17	1635 (1)	1395 (1)
	18–39	6002 (3)	8150 (5)
	40–64	40,181 (20)	34,603 (22)
	65–79	117,631 (60)	82,330 (52)
	≥80	31,926 (16)	31,486 (20)
	Mean (SD)^b^	67 (14)	66 (16)
Year of diagnosis			
	2004–2009	62,183 (32)	48,974 (31)
	2010–2014	59,633 (30)	46,789 (30)
	2015–2020	75,559 (38)	62,201 (39)
Person years			
		49,876,277 (50)	49,956,721 (50)
Cohabitation status^c^			
	Living with a partner	130,533 (71)	80,899 (56)
	Living alone	53,708 (29)	63,909 (44)
Education^c^			
	Short	62,784 (34)	64,643 (45)
	Medium	83,962 (46)	50,143 (34)
	Long	37,495 (20)	30,022 (21)
Charlson Comorbidity Index (CCI)^c^			
	0	136,107 (74)	112,958 (78)
	1	28,377 (15)	21,086 (15)
	2+	19,757 (11)	10,764 (7)
Cancer site			
*Head and neck*	Oral cavity	3640 (2)	2034 (1)
	Salivary glands	433 (0)	395 (0)
	Oropharynx	2959 (1)	1025 (1)
	Other pharynx	1338 (1)	336 (1)
	Thyroid	1179 (1)	3077 (2)
*Digestive organs*	Oesophagus	4960 (3)	1791 (1)
	Stomach	5509 (3)	2743 (2)
	Small intestine	1009 (1)	900 (1)
	Colon	21,515 (11)	21,534 (14)
	Rectal	13,265 (7)	8444 (5)
	Anal	561 (0)	1308 (1)
	Liver	3764 (2)	1609 (1)
	Gallbladder and biliary tract	1220 (1)	1605 (1)
	Gallbladder (excluding biliary tract)^d^	206 (0)	532 (0)
	Pancreas	6905 (4)	6636 (4)
*Respiratory*	Nasal cavity	544 (0)	378 (0)
	Larynx	2891 (2)	622 (0)
	Lung and trachea	31,256 (16)	30,141 (19)
	Pleura	1425 (1)	263 (0)
*Bone and connective tissue*	Bone and cartilage	502 (0)	363 (0)
	Connective tissue	1673 (1)	1567 (1)
*Skin*	Malignant melanoma	14,306 (7)	16,903 (11)
	Non-melanoma	18,252 (9)	14,243 (9)
*Urinary tract*	Kidney	7387 (4)	3825 (2)
	Renal pelvis	1423 (1)	935 (1)
	Bladder	17,901 (9)	5949 (4)
*Central nervous system*	Eye	669 (0)	669 (0)
	Meninges	1883 (1)	5439 (3)
	Brain and CNS	9260 (5)	8358 (5)
*Haematological*	Hodgkin lymphoma	1216 (1)	914 (1)
	Non-Hodgkin lymphoma	9557 (5)	7439 (5)
	Multiple myeloma	3405 (2)	2596 (2)
	Acute lymphocytic leukaemia	605 (0)	437 (0)
	Acute myeloid leukaemia	1519 (1)	1223 (1)
	Chronic lymphocytic leukaemia	3364 (2)	2100 (1)
	Chronic myeloid leukaemia	502 (0)	382 (0)
*All cancers combined*^*e*^		197,375 (56)	157,964 (44)

^a^Number and proportion (%). Percentages are rounded to whole numbers.

^b^Standard deviation.

^c^Restricted to individuals aged $\ge$30 years at diagnosis, born after 1920, not immigrated <5 years prior to diagnosis, and no missing information on cohabitation status and education level.

^d^Excluding C24 (Other and unspecified parts of the biliary tract).

^e^Defined as any of the 35 examined non-sex-specific cancer sites.

For all examined cancers combined, the IRR_M:F_ was 1.52 (95% CI 1.51–1.53). Age-standardised incidence rates were higher for males than females for the majority of individual cancers, with IRDs_M-F_ ranging from -9.7 (95% CI −10.9 to −8.5) for meningeal tumours to 41.3 (95% CI 39.0–43.6) for bladder cancer. IRRs_M:F_ were increased for 24 of the 35 cancer sites, and numerically elevated for another 4 sites, but the confidence intervals included the null. Incidences were higher in females than in males for thyroid and anal cancers, malignant melanoma, and meningeal tumours. For the four remaining cancer types, cancers of the gallbladder, bone and cartilage, eye and acute lymphocytic leukaemia, no differences in incidence between males and females were seen (Table 2). Although, in a sensitivity analyses, excluding other and unspecified parts of the biliary tract (C24), we observed numerically lower IRR_M:F_ for cancers of the gallbladder (0.89 95% CI 0.75–1.04) compared to 1.00 (95% CI 0.93–1.08) in the main analysis (Table 2). The lifetime risk of developing any of the examined cancers before age 100 was 35.1% in males and 28.9% in females, indicating that approximately one in six cancer cases in males could be avoided if they had the same risk as females.

**Table 2:** Incidence rates (IRs), rate differences (IRDs), incidence rate ratios (IRRs), relative survival ratios (RSs), relative survival differences (RSDs) and excess mortality ratios (EMRs) with 95% confidence intervals (CIs) for 35 non-sex-specific cancer sites, comparing males with females.

		Incidence				Survival and excess mortality			
		Males	Females			Males	Females		
	Cancer site	IR^a^ (95% CI)^b^	IR^a^ (95% CI)^b^	IRD^c^ (95% CI)^b^	IRR^d^ (95% CI)^b^	RS^e^ (95% CI)^b^	RS^e^ (95% CI)^b^	RSD^f^ (%-units)^e^ (95% CI)^b^	EMR^g^ (95% CI)^b^
Head and neck	Oral cavity	16.1 (15.6 to 16.6)	9.8 (9.4 to 10.2)	6.3 (5.0 to 7.6)	1.88 (1.78 to 1.99)	55.4 (53.5 to 57.3)	63 (60.4 to 65.6)	−7.6 (−13.9 to −1.3)	1.27 (1.15 to 1.41)
	Salivary glands	4.7 (4.3 to 5.2)	3.9 (3.5 to 4.3)	0.8 (−0.4 to 2.0)	1.12 (0.97 to 1.28)	63.1 (57.6 to 69.1)	79.8 (74.7 to 85.2)	−16.7 (−32.0 to −1.4)	1.99 (1.45 to 2.75)
	Oropharynx	7.6 (7.2 to 8.0)	4.6 (4.2 to 5.1)	3.0 (1.8 to 4.2)	1.70 (1.52 to 1.91)	56.4 (53.1 to 59.9)	53.9 (48.6 to 59.8)	2.5 (−10.3 to 15.3)	1.08 (0.95 to 1.23)
	Other pharynx	14.5 (14.0 to 15.1)	6.0 (5.6 to 6.4)	8.5 (7.2 to 9.8)	2.58 (2.40 to 2.77)	51.6 (49.6 to 53.6)	59.7 (56.3 to 63.3)	−8.1 (−16.0 to −0.2)	1.25 (1.06 to 1.48)
	Thyroid	5.3 (5.0 to 5.6)	9.3 (9.0 to 9.6)	−4.0 (−4.8 to −3.2)	0.56 (0.52 to 0.59)	84.7 (82.2 to 87.4)	91.8 (90.6 to 93.1)	−7.1 (−12.8 to −1.4)	1.86 (1.48 to 2.33)
Digestive organs	Oesophagus	24.6 (23.9 to 25.3)	10.7 (10.2 to 11.3)	13.9 (12.2 to 15.6)	2.86 (2.71 to 3.02)	14.3 (13.2 to 15.5)	15.3 (13.4 to 17.3)	−1 (−5.4 to 3.4)	1.03 (0.97 to 1.10)
	Stomach	23.5 (22.8 to 24.1)	12.5 (12.0 to 13.0)	11.0 (9.4 to 12.6)	2.25 (2.15 to 2.36)	21.6 (20.4 to 23.0)	22.8 (21.0 to 24.7)	−1.2 (−5.6 to 3.2)	1.01 (0.95 to 1.07)
	Small intestine	7.1 (6.7 to 7.6)	6.4 (6.0 to 6.8)	0.7 (−0.5 to 1.9)	1.20 (1.10 to 1.31)	53.4 (49.8 to 57.3)	55.2 (51.4 to 59.3)	−1.8 (−12.5 to 8.9)	1.07 (0.92 to 1.24)
	Colon	72.0 (71.1 to 73.0)	68.5 (67.6 to 69.5)	3.5 (0.9 to 6.1)	1.27 (1.25 to 1.30)	61.0 (60.1 to 61.8)	61.6 (60.7 to 62.4)	−0.6 (−3.0 to 1.8)	1.02 (0.99 to 1.06)
	Rectal	51.5 (50.6 to 52.4)	31.8 (31.1 to 32.5)	19.7 (17.5 to 21.9)	1.91 (1.86 to 1.96)	63.5 (62.5 to 64.6)	65.4 (64.2 to 66.7)	−1.9 (−5.1 to 1.3)	1.08 (1.02 to 1.13)
	Anal	5.0 (4.6 to 5.4)	7.1 (6.7 to 7.5)	−2.1 (−3.2 to −1.0)	0.72 (0.66 to 0.80)	62.5 (57.9 to 67.5)	73.8 (70.9 to 76.8)	−11.3 (−22.3 to −0.3)	1.56 (1.28 to 1.90)
	Liver	17.9 (17.3 to 18.5)	8.9 (8.4 to 9.3)	9.0 (7.5 to 10.5)	2.40 (2.26 to 2.54)	8.1 (7.1 to 9.2)	10.4 (8.8 to 12.3)	−2.3 (−6.3 to 1.7)	1.05 (0.99 to 1.12)
	Gallbladder and biliary tract	8.5 (8.0 to 9.0)	10.2 (9.7 to 10.7)	−1.7 (−3.1 to −0.3)	1.00 (0.93 to 1.08)	13.4 (11.3 to 15.8)	10.5 (8.9 to 12.4)	2.9 (−2.7 to 8.5)	0.91 (0.84 to 0.99)
	Gallbladder (excluding biliary tract)^h^	5.2 (4.5 to 6.0)	6.4 (5.8 to 6.9)	−1.2 (−3.0 to 0.6)	0.89 (0.75 to 1.04)	14.5 (9.8 to 21.4)	11.9 (9.1 to 15.7)	2.6 (−10.5 to 15.7)	0.98 (0.82 to 1.18)
	Pancreas	30.4 (29.7 to 31.1)	28.3 (27.6 to 29.0)	2.1 (0.2 to 4.0)	1.30 (1.26 to 1.34)	6.5 (5.9 to 7.3)	6.3 (5.7 to 7.1)	0.2 (−1.7 to 2.1)	1.05 (1.01 to 1.09)
Respiratory	Nasal cavity	5.2 (4.7 to 5.6)	4.6 (4.1 to 5.1)	0.6 (−0.7 to 1.9)	1.18 (1.04 to 1.35)	57.0 (52.2 to 62.2)	59.8 (54.1 to 66.1)	−2.8 (−18.1 to 12.5)	1.18 (0.93 to 1.49)
	Larynx	15.2 (14.7 to 15.8)	5.5 (5.1 to 6.0)	9.7 (8.3 to 11.1)	2.96 (2.72 to 3.23)	62.8 (60.7 to 64.9)	62.4 (58.1 to 67.0)	0.4 (−9.2 to 10.0)	0.97 (0.83 to 1.14)
	Lung and trachea	122.4 (121.1 to 123.8)	108.7 (107.4 to 109.9)	13.7 (10.1 to 17.3)	1.28 (1.26 to 1.30)	13.5 (13.1 to 14.0)	18.9 (18.4 to 19.4)	−5.4 (−6.7 to −4.1)	1.17 (1.15 to 1.20)
	Pleura	11.0 (10.5 to 11.6)	4.7 (4.2 to 5.3)	6.3 (4.8 to 7.8)	2.61 (2.29 to 2.98)	6.0 (4.6 to 7.7)	9.3 (6.0 to 14.4)	−3.3 (−12.1 to 5.5)	1.14 (0.98 to 1.33)
Bone and connective tissue	Bone and cartilage	3.6 (3.3 to 3.9)	3.4 (3.1 to 3.8)	0.2 (−0.7 to 1.1)	1.03 (0.90 to 1.18)	67.3 (62.8 to 72.2)	75.1 (70.3 to 80.2)	−7.8 (−21.2 to 5.6)	1.26 (0.95 to 1.69)
	Connective tissue	6.7 (6.4 to 7.0)	6.2 (5.9 to 6.5)	0.5 (−0.3 to 1.3)	1.14 (1.06 to 1.22)	62.1 (59.4 to 65.0)	54.4 (51.6 to 57.4)	7.7 (−0.2 to 15.6)	0.79 (0.70 to 0.89)
Skin	Malignant melanoma	38.9 (38.3 to 39.6)	42.8 (42.2 to 43.5)	−3.9 (−5.7 to −2.1)	0.95 (0.92 to 0.97)	88.3 (87.5 to 89.1)	94.1 (93.6 to 94.7)	−5.8 (−7.7 to −3.9)	1.86 (1.67 to 2.08)
	Non-melanoma	69.1 (68.1 to 70.1)	50.6 (49.7 to 51.4)	18.5 (15.9 to 21.1)	1.78 (1.75 to 1.82)	89.6 (88.6 to 90.7)	94.9 (93.8 to 96.1)	−5.3 (−8.3 to −2.2)	1.95 (1.64 to 2.33)
Urinary tract	Kidney	26.1 (25.5 to 26.7)	14.5 (14.1 to 15.0)	11.6 (10.1 to 13.1)	2.09 (2.01 to 2.17)	63.3 (62.0 to 64.7)	63.2 (61.4 to 65.1)	0.1 (−4.4 to 4.6)	1.08 (1.00 to 1.16)
	Renal pelvis	9.9 (9.4 to 10.5)	8.0 (7.5 to 8.5)	1.9 (0.4 to 3.4)	1.49 (1.37 to 1.62)	57.7 (54.7 to 60.9)	52 (48.3 to 55.9)	5.7 (−3.9 to 15.3)	0.89 (0.78 to 1.02)
	Bladder	67.4 (66.4 to 68.4)	26.1 (25.5 to 26.8)	41.3 (39.0 to 43.6)	3.65 (3.55 to 3.76)	70.5 (69.6 to 71.4)	63.6 (62.1 to 65.1)	6.9 (3.5 to 10.3)	0.72 (0.68 to 0.77)
Central nervous system	Eye	4.8 (4.4 to 5.2)	4.8 (4.5 to 5.2)	0.0 (−1.0 to 1.0)	1.04 (0.93 to 1.16)	77.4 (73.4 to 81.6)	81.5 (77.7 to 85.4)	−4.1 (−15.1 to 6.9)	1.38 (1.03 to 1.86)
	Meninges	8.0 (7.6 to 8.4)	17.7 (17.3 to 18.2)	−9.7 (−10.9 to −8.5)	0.45 (0.43 to 0.48)	88.6 (86.4 to 90.8)	92.9 (91.8 to 94.0)	−4.3 (−9.1 to 0.5)	1.71 (1.35 to 2.15)
	Brain	20.2 (19.7 to 20.6)	18.3 (17.9 to 18.7)	1.9 (0.7 to 3.1)	1.18 (1.15 to 1.22)	58.9 (57.7 to 60.0)	66.1 (65.0 to 67.3)	−7.2 (−10.4 to −4.0)	1.24 (1.18 to 1.31)
Haematological	Hodgkin lymphoma	4.5 (4.3 to 4.8)	4.2 (3.9 to 4.5)	0.3 (−0.5 to 1.1)	1.07 (0.98 to 1.16)	87.8 (85.6 to 90.0)	87.9 (85.5 to 90.4)	−0.1 (−6.5 to 6.3)	0.98 (0.75 to 1.30)
	Non-Hodgkin lymphoma	26.1 (25.6 to 26.7)	22.9 (22.3 to 23.4)	3.2 (1.7 to 4.7)	1.47 (1.42 to 1.51)	71.9 (70.8 to 73.1)	75.4 (74.2 to 76.7)	−3.5 (−6.8 to −0.2)	1.25 (1.16 to 1.34)
	Multiple myeloma	16.6 (16.0 to 17.1)	13.0 (12.5 to 13.5)	3.6 (2.1 to 5.1)	1.50 (1.43 to 1.58)	52.5 (50.4 to 54.7)	55.5 (53.1 to 58.0)	−3 (−9.4 to 3.4)	1.20 (1.10 to 1.32)
	Acute lymphocytic leukaemia	4.2 (3.9 to 4.5)	4.3 (3.9 to 4.7)	−0.1 (−1.1 to 0.9)	0.98 (0.87 to 1.11)	77.3 (73.8 to 81.0)	73.1 (68.8 to 77.6)	4.2 (−6.9 to 15.3)	0.99 (0.76 to 1.29)
	Acute myeloid leukaemia	7.3 (7.0 to 7.7)	6.1 (5.7 to 6.4)	1.2 (0.2 to 2.2)	1.27 (1.18 to 1.37)	24.9 (22.5 to 27.4)	27.9 (25.3 to 30.8)	−3 (−10.2 to 4.2)	0.97 (0.89 to 1.07)
	Chronic lymphocytic leukaemia	17.3 (16.7 to 17.9)	11.9 (11.4 to 12.4)	5.4 (3.9 to 6.9)	1.78 (1.69 to 1.88)	84.5 (82.6 to 86.4)	89 (86.8 to 91.2)	−4.5 (−10.2 to 1.2)	1.72 (1.37 to 2.16)
	Chronic myeloid leukaemia	4.0 (3.6 to 4.3)	3.9 (3.5 to 4.3)	0.1 (−0.9 to 1.1)	1.10 (0.96 to 1.26)	80.5 (76.3 to 84.9)	81.8 (77.3 to 86.7)	−1.3 (−13.8 to 11.2)	1.19 (0.83 to 1.72)
All cancers combined		401.4 (399.6 to 403.2)	318.1 (316.6 to 319.7)	83.3 (78.6 to 88.0)	1.52 (1.51 to 1.53)	54.1 (53.8 to 54.4)	58.3 (58.0 to 58.6)	−4.2 (−5.0 to −3.4)	1.09 (1.08 to 1.10)

^a^Incidence rate per 100,000 person-years age-standardised to the Danish population in 2010.

^b^Corresponding to 95% confidence intervals.

^c^Incidence rate-difference per 100,000 person-years.

^d^Incidence rate ratio comparing males to females. Adjusted for age and year of diagnosis.

^e^Relative survival up to 5 years from diagnosis.

^f^Relative survival rate-difference in %-units up to 5 years from diagnosis.

^g^Excess mortality ratio comparing males to females, with follow-up up to 5 years after diagnosis. Adjusted for age and year of diagnosis.

^h^Excluding C24 (Other and unspecified parts of the biliary tract).

For all examined cancers combined, RS was lower among males (54.1, 95% CI 53.8–54.4) than females (58.3, 95% CI 58.0–58.6), yielding an EMR_M:F_ of 1.09 (95% CI 1.08–1.10) (Table 2). Across the 35 cancer sites, lower survival in males (EMRs_M:F_ > 1) was observed for 16 sites. For additional 7 sites, the point estimates suggested higher mortality in males, but the confidence intervals included the null. EMRs_M:F_ was less than one in four sites, i.e. cancers of the gallbladder, connective tissues, bladder and renal pelvis (the confidence interval included the null for the latter). No differences in survival were seen for cancers of the oropharynx, oesophagus, stomach, colon, larynx, Hodgkin lymphoma, acute lymphocytic leukaemia and acute myeloid leukaemia (Table 2). The lifetime risk of dying from any of the examined cancers before age 100 years was 19.5% in males and 15.3% in females, suggesting that one in five cancer deaths in males could be avoided if they had the same risk as females.

**Fig. 1:**
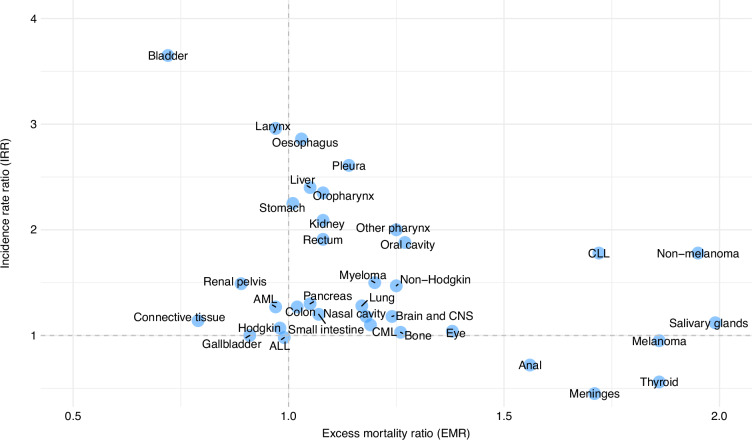
IRRs by EMRs for 35 non-sex-specific cancer sites, comparing males with females. All estimates are adjusted for age and year of diagnosis. ALL acute lymphocytic leukaemia, AML acute myeloid leukaemia, CLL chronic lymphocytic leukaemia, CML chronic myeloid leukaemia.

In age-stratified analyses, the EMRs_M:F_ were greater at younger ages for most cancer sites. For all cancers combined, the EMR_M:F_ was 1.22 (95% CI 1.20–1.25) among individuals aged 18–64 years, compared with 1.01 (95% CI 0.99–1.02) among those aged 65–99 years (Supplementary Table 5). When using age 50 as cut point the difference was even more pronounced with an EMR_M:F_ of 1.45 (95% CI 1.38–1.52) among individuals aged 18–49 years, compared with 1.06 (95% CI 1.05–1.07) among those aged 50–99 years (Supplementary Table 6). The relationship between incidence and mortality, comparing males to females, is illustrated in Fig. 1.

### Aetiological groups

**Fig. 2:**
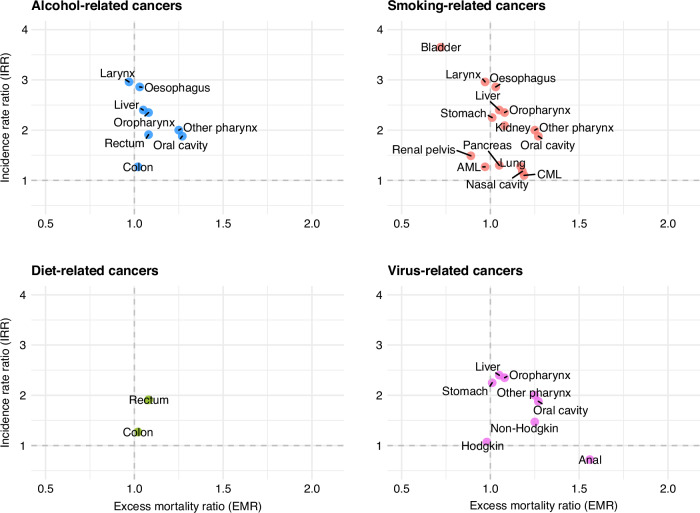
IRRs by EMRs for non-sex-specific cancers aetiologically related to alcohol, smoking, diet and/or viruses, comparing males with females. All estimates are adjusted for age and year of diagnosis. AML acute myeloid leukaemia, CML chronic myeloid leukaemia.

The age-adjusted incidence of most cancers related to alcohol and smoking was more than 1.5-fold higher in males than females (Fig. 2). IRRs_M:F_ were elevated across all cancers related to alcohol and diet, in 14 out of 15 smoking-related cancers and in 6 out of 8 virus-related cancers. IRR_M:F_ was decreased for one virus-related cancer (anal cancer). EMRs_M:F_ were elevated in 3 out of 8 alcohol-related cancers, 4 out of 15 smoking-related cancers, 1 out of 2 diet-related cancers and in 4 out of 8 virus-related cancers, whereas EMR_M:F_ was decreased for only one smoking-related cancer (bladder cancer).

### Analysis of the modifying effects of cohabitation, education and comorbidity

**Fig. 3:**
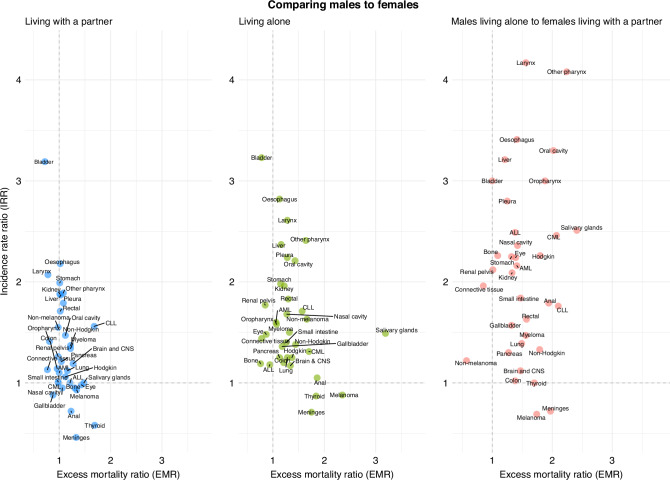
IRRs by EMRs for 35 non-sex-specific cancer sites stratified by cohabitation status, comparing males with females. Study population restricted to individuals aged ≥30 years, born after 1920, not immigrated <5 years prior to diagnosis, and no missing information on cohabitation status or education level. All estimates are adjusted for age and year of diagnosis. ALL acute lymphocytic leukaemia, AML acute myeloid leukaemia, CLL chronic lymphocytic leukaemia, CML chronic myeloid leukaemia.

In analyses stratified according to cohabitation status, IRRs_M:F_ and EMRs_M:F_ were increased both among individuals living alone and individuals living with a partner (Fig. 3). Effect modification analyses demonstrated elevated male susceptibility to the effects of living alone for all cancers combined (Supplementary Fig. 2), and on the multiplicative scale for 12 of 35 cancers, RRs ranging from 1.13 (95% CI 1.08–1.17) for lung and trachea to 1.78 (95% CI 1.02–3.11) for salivary glands. On the additive scale, male susceptibility was elevated for 21 of 35 cancers, RERIs ranging from 0.09 (95% CI 0.01–0.18) for pancreas to 1.21 (95% CI 0.35–2.06) for salivary glands. To illustrate the scope of the combined effects of being male and living alone, we plotted IRRs_M:F_ and EMRs_M:F_ comparing males living alone to females living with a partner. This comparison showed even higher IRRs_M:F_ and EMRs_M:F_ for most cancers, especially for cancers related to alcohol and smoking (Fig. 3 and Supplementary Fig. 3).

For all cancers combined, the effect of medium education seemed to have a greater negative impact on survival in males than in females (RERI: 0.11 (95% CI 0.07–0.14)). However, no effect modification was seen for short education (Supplementary Fig. 2). When examining by cancer type, cancers of the salivary glands, colon, gallbladder and kidney having medium or short education had a greater negative impact on survival in males compared with females. In contrast, shorter education than long had greater negative impact on survival in females with cancers in pancreas and bone and cartilage.

For the combined group of examined cancers the comorbidity burden was higher in males than in females, reflecting similar uneven distributions for the vast majority of the individual cancers (Supplementary Table 7). This was supported by a chi-squared test comparing the distribution of CCI levels between sexes (*χ*^2^test, *p* < 0.005). For all cancers combined, comorbidity was associated with a greater negative effect on survival in females than males (RERI: CCI 2+−0.04 (95% CI −0.10 to −0.02)). For individual cancer types, comorbidity had a stronger impact in males compared with females for kidney cancer and a stronger impact in females compared with males in cancers of the oesophagus, stomach, rectal, liver and connective tissue (Supplementary Fig. 2).

## Discussion

We compared incidence and survival between males and females for 35 non-sex-specific cancers and found that males had more than 50% higher risk of developing any of these cancers and upon cancer diagnosis approximately 10% higher cancer-related mortality than females. This male cancer disadvantage was not limited to specific cancers but rather reflective of a general trend: compared with females, males had higher incidence and lower relative survival for 24 and 16 of the 35 cancer types, respectively. In contrast, for females the corresponding numbers were 4 and 3 out of the 35 cancers investigated. We estimated that one in six cancers and one in five cancer deaths in males would be avoided if they had the same lifetime risk of cancer incidence and mortality as females in our cohort. Finally, our results revealed that males living alone are a particularly vulnerable subgroup with high cancer incidence and poor survival.

Our findings are consistent with previous studies in comparable settings [4–7, 26], which emphasise that males seem continually to be at a disadvantage in terms of both cancer risk and survival when compared with females. Our findings may even be a conservative illustration of this disadvantage. Even for common cancers such as colon cancer, where males and females had similar relative survival, the life-years lost would be greater in males owing to a higher incidence. Likewise, while males clearly had higher relative survival after bladder cancer than females, the absolute number of deaths was still higher among males (6705) than females (2477) due to their higher incidence.

Sex differences in cancer likely result from a complex interplay between both biological and gender-related factors. The wide variety of cancers for which males had higher incidence and/or mortality may indicate the existence of fundamental biological mechanisms which have been suggested to include e.g. differences in sex chromosomes and hormones, immune system activity and metabolism [8]. Consistent with this, we observed greater sex differences in survival among individuals diagnosed before the age of 50 years, possibly reflecting a protective effect of female hormones that declines with advancing age [26]. However, due to the register-based nature of our study, we could not disentangle the impact of biological factors on cancer incidence and survival.

Gender-related factors such as socioeconomic factors and health behaviours like higher alcohol consumption and smoking rates in males, contribute to their higher cancer burden [13, 27]. Unhealthy lifestyles could also explain the higher prevalence of comorbidities at diagnosis, impacting male cancer mortality. Strongly supporting this, our analyses revealed that males had both higher incidence and lower survival than females for nearly all cancers linked to unhealthy lifestyles, including alcohol consumption, smoking and dietary patterns, with the greatest male disadvantage in incidence seen in oesophageal, larynx and bladder cancers. Variations in health literacy and healthcare utilisation may also contribute to the cancer disadvantage in males. Males are often less inclined than women to discuss health concerns and may be less likely to seek medical attention [28, 29]. Together, these factors likely contribute to delayed diagnosis, advanced disease stage and worse outcomes in males [4, 5, 14, 26].

Strikingly, we found that living alone had a greater negative effect on survival in males than females, reflecting broader patterns of cohabitation. Males living alone may receive less emotional support which could impact their health behaviour (i.e. smoking and alcohol use as well as diet), health literacy (e.g. through validation of symptoms within their social network), and healthcare utilisation (e.g. through emotional support and practical assistance in seeking care) [29]. These factors correlate with circumstances of living alone, and apparently more so in males than in females. For instance, it has been documented that males living alone participate less in colorectal screening [11], and are more likely to be diagnosed at a more advanced stage when diagnosed with head and neck cancers compared to those with a partner. This pattern was not observed for females living alone [14]. These mechanisms add to the lower survival observed for most cancers among males living alone.

The general picture of a male cancer disadvantage notwithstanding, we would be amiss not to mention that females had poorer survival rates for gallbladder, connective tissue and bladder cancers. This may indicate that females with these cancers may face delayed diagnosis and/or receive less effective treatment compared to males [3].

### Strengths and limitations of this study

A major strength of this study is the large sample size, comprising practically all males and females living in Denmark from 2004 to 2020. The generalisability of these findings to similar countries is high due to the comprehensive nature of the data and the minimal loss to follow-up. Through Danish population-based registers, with no influence by self-reports and thus low risk of information bias, we obtained information on cohabitation status, education level and comorbidity, leveraging the possibility to assess whether these factors modified sex differences in cancer survival, which, to our knowledge, no previous study have explored.

Although our analyses are based on high-quality register data and robust statistical methods, some limitations must be noted. First, our ability to disentangle biological mechanisms was limited, restricting our assessment of sex-based differences in cancer risk and survival due to biological factors. Second, although living with a partner included same sex couples who were married or in registered partnerships, the available data did not allow us to identify unregistered same-sex couples, and they were thus categorised as living alone. Cohabitation status was entered as a time-dependent variable updated once a year in the incidence analyses, whereas in the survival analyses, cohabitation status was defined as that on 31 December of the year preceding cancer diagnosis. These definitions may have led to some misclassification of cohabitation status. Third, the lack of information on individual-level health behaviour constrains our ability to directly evaluate the contribution of factors such as smoking, alcohol consumption and diet to the observed sex differences. However, by grouping cancers based on aetiological risk factors, we provide some insight into how these factors may influence sex-specific differences in cancer. Additionally, we did not account for differences in stage at diagnosis and treatment, preventing us from assessing whether these factors may explain some of the observed sex differences. Last, we could not explore the impact of gender identity due to the use of register-based data, which limited us to a binary sex classification. We acknowledge the importance of non-binary identities [30] and the need for future research to address this.

## Conclusion

Males had higher incidence and mortality than females across most of the studied cancers. Incidence and mortality were particularly increased for males with cancers related to alcohol and smoking, as well as among males living alone. The strong impact of sex on both incidence and survival indicates that there is a need for a gender-focused approach that can effectively enhance health behaviour, the use of healthcare services, diagnostics and treatment. Specifically, interventions directed towards males living alone may offer substantial benefits.

## Supplementary information

Supplementary

## Data Availability

The data utilised in this study were accessed remotely through the Danish Cancer Institute’s secure server at Statistics Denmark. Any access to data requires permission from the Danish Cancer Institute.

## Code availability

The code used to generate the results reported in this study can be accessed through the Danish Cancer Institute’s secure server at statistics Denmark. To get access to the code, contact the corresponding author Fie Stegenborg. E-mail: fiea@cancer.dk.
