# Supplementary material for: Sex differences in cancer incidence and survival: a Danish nationwide population-based study assessing 35 cancer sites
Source: Br J Cancer. 2026 Apr 27;135(2):273–82. doi: 10.1038/s41416-026-03429-7 (PMC13310841; doi:10.1038/s41416-026-03429-7)
Supplement: Supplementary file 1 — Supplementary [file 41416_2026_3429_MOESM1_ESM.pdf]

Supplementary

Figure 1: Flowchart of Danish males and females diagnosed with cancer in 2004-2020 included in the analyses 2

Figure 2: Relative risks (RRs) of death with 95% confidence intervals (CIs) for females and males, and measures of effect modification on the multiplicative scale (RR) and the additive scale (relative excess risk due to interaction [RERI]), by cohabitation status, education, and comorbidity (CCI), for 35 non-sex-specific cancer sites 3

Figure 3: IRRs by EMRs comparing males with females aged ≥ 30 years at diagnosis, for cancers related to alcohol and/or smoking, stratified by cohabitation status 15

Table 1: Classification of cancer sites 16

Table 2: Categorisation of cancer sites into etiological groups based on classifications from the International Agency for Research on Cancer (IARC) 18

Table 3: Individuals included in the effect modification analyses 20

Table 4: Number of cancer cases (n), proportions (%), number of events, person-years, and mean age at diagnosis for 35 non-sex-specific cancer sites, for males and females 21

Table 5: Excess mortality ratios (EMRs) with 95% confidence intervals (CI) comparing males with females for 35 non-sex-specific cancer sites, by age groups 18-64 and 65-99, respectively 23

Table 6: Excess mortality ratios (EMRs) with 95% confidence intervals (CI) comparing males with females for 35 non-sex-specific cancer sites, by age groups 18-49 and 50-99, respectively 24

Table 7: Number (n), proportions (%), number of events, and person-years by cohabitation status, education level, and comorbidity (CCI) for 35 non-sex-specific cancer sites, for males and females included in the effect modification analyses 25

STROBE Statement—checklist of items that should be included in reports of observational studies . 36

Figure 1: Flowchart of Danish males and females diagnosed with cancer in 2004-2020 included in the analyses

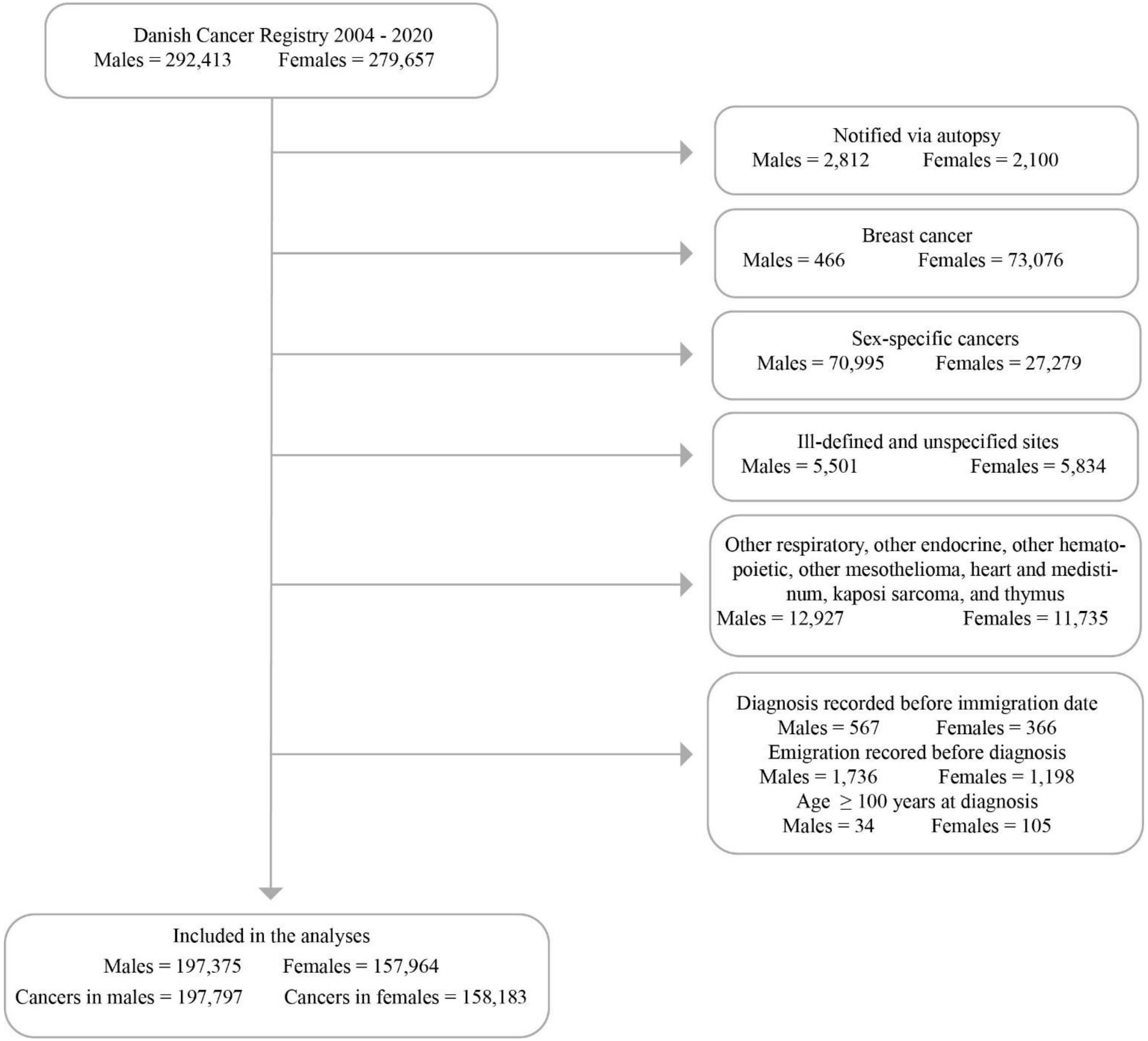

Figure 2: Relative risks (RRs) of death with 95% confidence intervals (CIs) for females and males, and measures of effect modification on the multiplicative scale (RR) and the additive scale (relative excess risk due to interaction [RERI]), by cohabitation status, education, and comorbidity (CCI), for 35 non-sex-specific cancer sites

CIs are not displayed in the figure when extending beyond the axis limits.

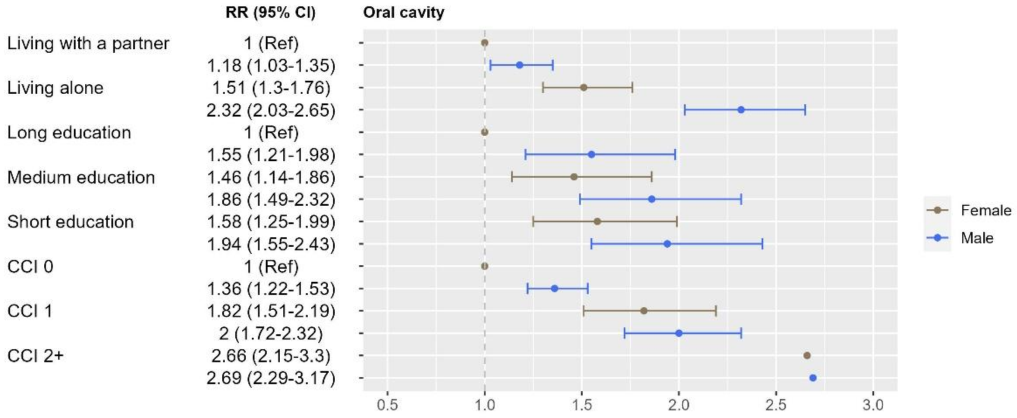

Measure of effect modification on multiplicative scale (RR) and additive scale (RERI):
Cohabitation status: RR (95% CI): 1.3 (1.08-1.56) | RERI (95% CI): 0.63 (0.38-0.88)
Medium education: RR (95% CI): 0.82 (0.62-1.1) | RERI (95% CI): -0.15 (-0.54-0.25)
Short education: RR (95% CI): 0.8 (0.6-1.05) | RERI (95% CI): -0.18 (-0.58-0.21)
CCI 1: RR (95% CI): 0.81 (0.64-1.01) | RERI (95% CI): -0.18 (-0.58-0.21)
CCI 2+: RR (95% CI): 0.74 (0.57-0.96) | RERI (95% CI): -0.34 (-0.98-0.31)

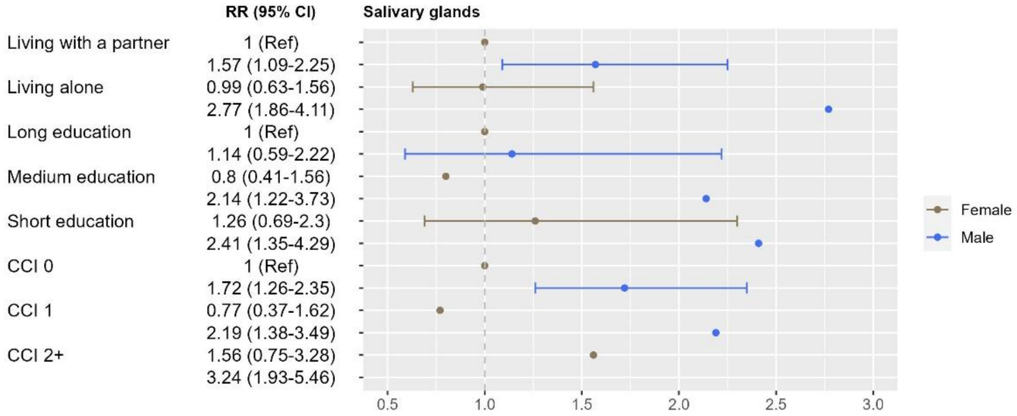

Measure of effect modification on multiplicative scale (RR) and additive scale (RERI):
Cohabitation status: RR (95% CI): 1.78 (1.02-3.11) | RERI (95% CI): 1.21 (0.35-2.06)
Medium education: RR (95% CI): 2.32 (1.02-5.28) | RERI (95% CI): 1.19 (0.4-1.97)
Short education: RR (95% CI): 1.66 (0.77-3.61) | RERI (95% CI): 1 (0.12-1.88)
CCI 1: RR (95% CI): 1.65 (0.7-3.89) | RERI (95% CI): 0.7 (-0.36-1.77)
CCI 2+: RR (95% CI): 1.21 (0.5-2.92) | RERI (95% CI): 0.96 (-0.89-2.82)

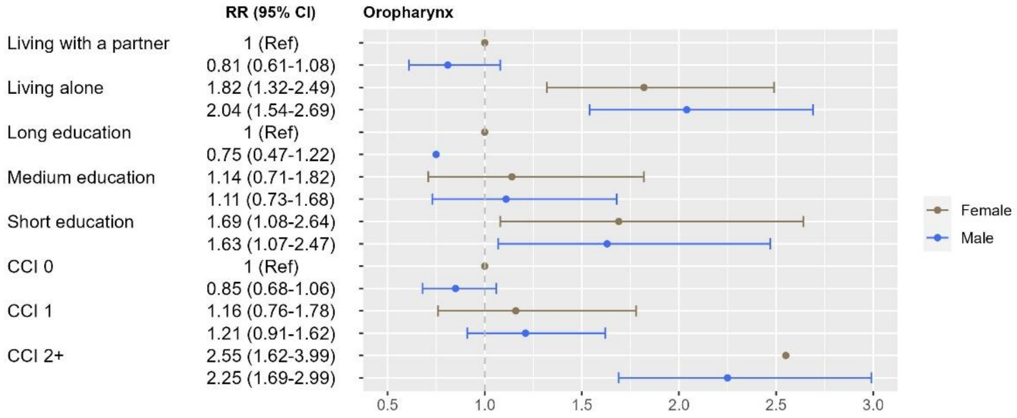

Measure of effect modification on multiplicative scale (RR) and additive scale (RERI):
Cohabitation status: RR (95% CI): 1.39 (0.96-2.01) | RERI (95% CI): 0.41 (-0.06-0.88)
Medium education: RR (95% CI): 1.29 (0.74-2.27) | RERI (95% CI): 0.22 (-0.28-0.72)
Short education: RR (95% CI): 1.28 (0.74-2.21) | RERI (95% CI): 0.19 (-0.39-0.76)
CCI 1: RR (95% CI): 1.23 (0.75-2.02) | RERI (95% CI): 0.2 (-0.35-0.75)
CCI 2+: RR (95% CI): 1.04 (0.62-1.74) | RERI (95% CI): -0.15 (-1.32-1.03)

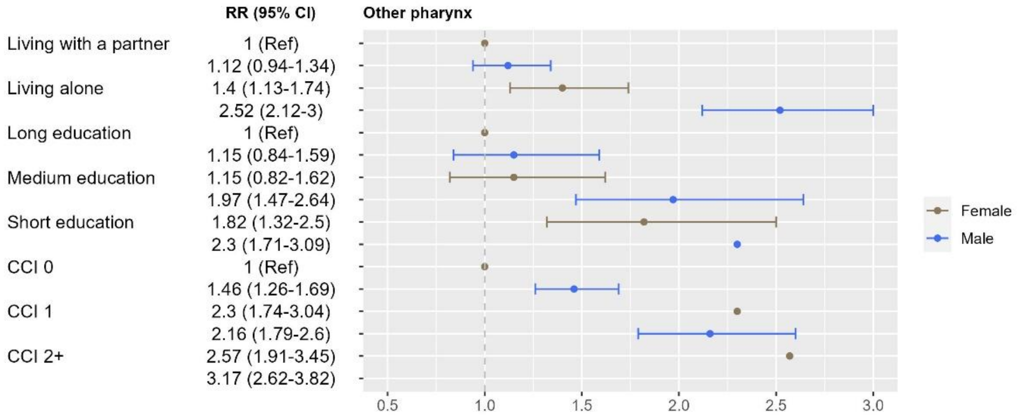

Measure of effect modification on multiplicative scale (RR) and additive scale (RERI):
Cohabitation status: RR (95% CI): 1.61 (1.27-2.04) | RERI (95% CI): 1 (0.72-1.29)
Medium education: RR (95% CI): 1.49 (1.02-2.17) | RERI (95% CI): 0.67 (0.34-0.99)
Short education: RR (95% CI): 1.1 (0.77-1.58) | RERI (95% CI): 0.33 (-0.09-0.75)
CCI 1: RR (95% CI): 0.64 (0.47-0.88) | RERI (95% CI): -0.61 (-1.28-0.07)
CCI 2+: RR (95% CI): 0.84 (0.61-1.17) | RERI (95% CI): 0.14 (-0.68-0.95)

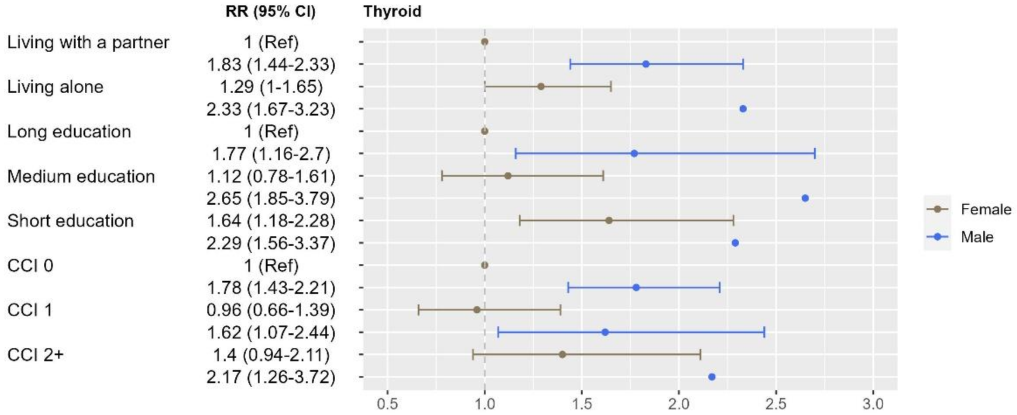

Measure of effect modification on multiplicative scale (RR) and additive scale (RERI):
Cohabitation status: RR (95% CI): 0.99 (0.65-1.49) | RERI (95% CI): 0.21 (-0.57-0.99)
Medium education: RR (95% CI): 1.33 (0.79-2.26) | RERI (95% CI): 0.76 (-0.1-1.61)
Short education: RR (95% CI): 0.79 (0.47-1.33) | RERI (95% CI): -0.12 (-1.03-0.79)
CCI 1: RR (95% CI): 0.95 (0.54-1.66) | RERI (95% CI): -0.12 (-0.9-0.66)
CCI 2+: RR (95% CI): 0.87 (0.44-1.71) | RERI (95% CI): -0.02 (-1.31-1.28)

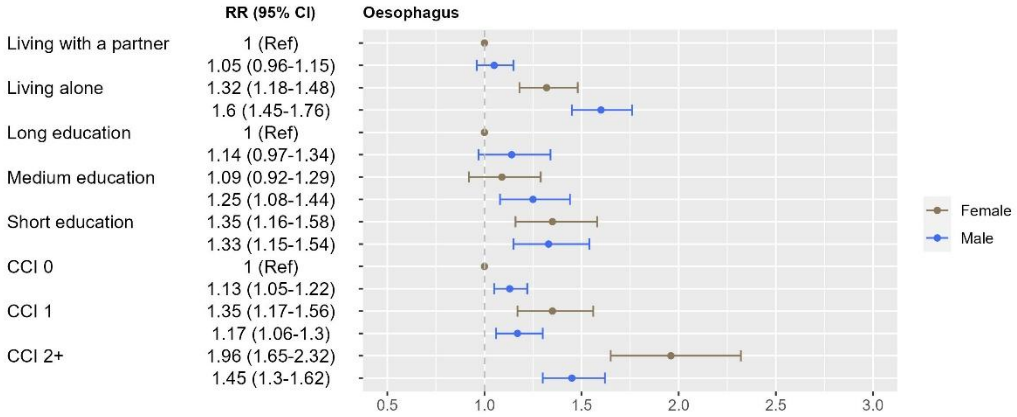

Measure of effect modification on multiplicative scale (RR) and additive scale (RERI):
Cohabitation status: RR (95% CI): 1.15 (1.01-1.31) | RERI (95% CI): 0.23 (0.08-0.38)
Medium education: RR (95% CI): 1 (0.83-1.22) | RERI (95% CI): 0.02 (-0.19-0.22)
Short education: RR (95% CI): 0.86 (0.72-1.04) | RERI (95% CI): -0.16 (-0.38-0.06)
CCI 1: RR (95% CI): 0.77 (0.65-0.91) | RERI (95% CI): -0.3 (-0.52--0.09)
CCI 2+: RR (95% CI): 0.66 (0.54-0.8) | RERI (95% CI): -0.63 (-0.99--0.28)

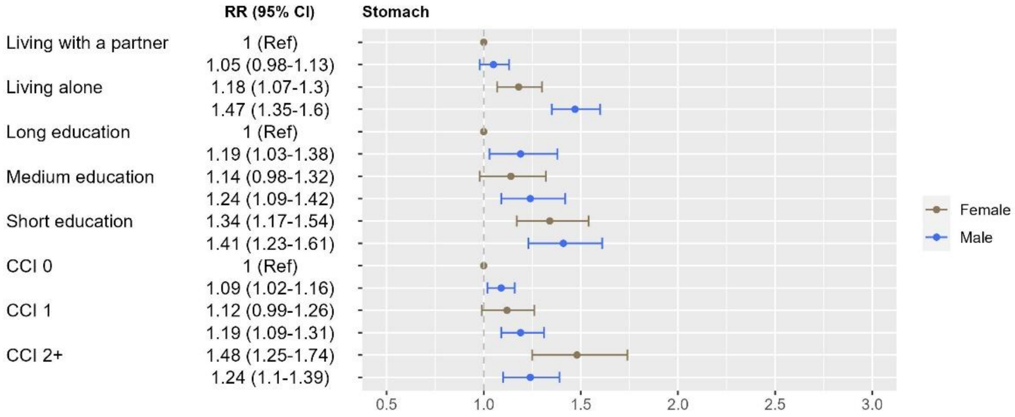

Measure of effect modification on multiplicative scale (RR) and additive scale (RERI):
Cohabitation status: RR (95% CI): 1.19 (1.06-1.33) | RERI (95% CI): 0.24 (0.11-0.37)
Medium education: RR (95% CI): 0.92 (0.77-1.09) | RERI (95% CI): -0.09 (-0.29-0.11)
Short education: RR (95% CI): 0.88 (0.75-1.04) | RERI (95% CI): -0.12 (-0.32-0.08)
CCI 1: RR (95% CI): 0.99 (0.85-1.15) | RERI (95% CI): -0.01 (-0.18-0.16)
CCI 2+: RR (95% CI): 0.77 (0.63-0.94) | RERI (95% CI): -0.33 (-0.6--0.05)

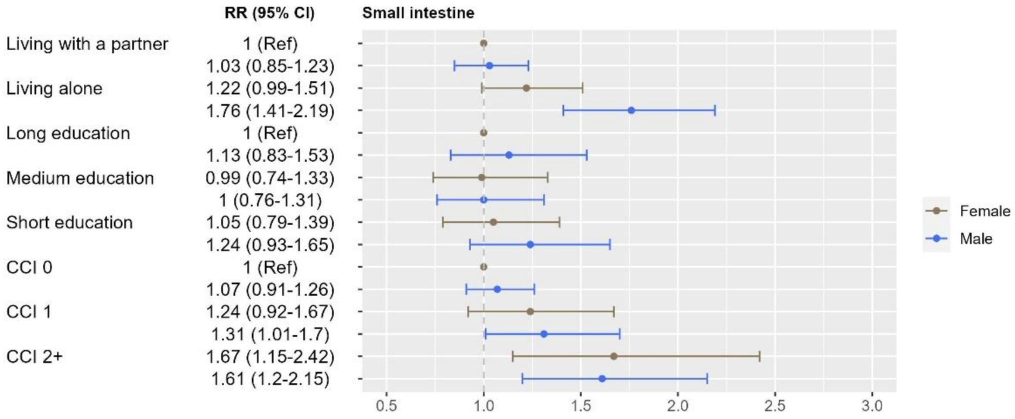

Measure of effect modification on multiplicative scale (RR) and additive scale (RERI):
Cohabitation status: RR (95% CI): 1.4 (1.04-1.89) | RERI (95% CI): 0.51 (0.13-0.89)
Medium education: RR (95% CI): 0.89 (0.61-1.31) | RERI (95% CI): -0.12 (-0.53-0.29)
Short education: RR (95% CI): 1.05 (0.72-1.54) | RERI (95% CI): 0.07 (-0.34-0.47)
CCI 1: RR (95% CI): 0.98 (0.66-1.46) | RERI (95% CI): 0 (-0.49-0.48)
CCI 2+: RR (95% CI): 0.9 (0.56-1.43) | RERI (95% CI): -0.14 (-0.89-0.61)

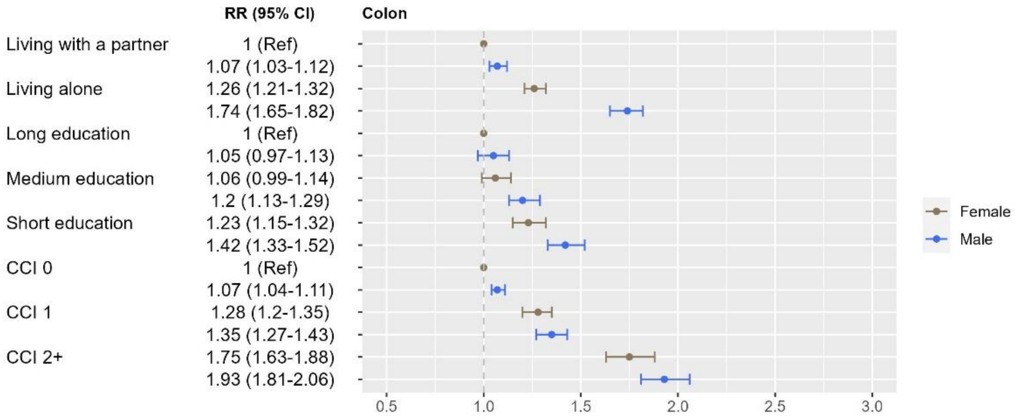

Measure of effect modification on multiplicative scale (RR) and additive scale (RERI):
Cohabitation status: RR (95% CI): 1.28 (1.2-1.37) | RERI (95% CI): 0.4 (0.32-0.48)
Medium education: RR (95% CI): 1.08 (0.98-1.18) | RERI (95% CI): 0.09 (0-0.18)
Short education: RR (95% CI): 1.1 (1.01-1.2) | RERI (95% CI): 0.14 (0.05-0.23)
CCI 1: RR (95% CI): 0.98 (0.91-1.07) | RERI (95% CI): 0 (-0.11-0.1)
CCI 2+: RR (95% CI): 1.03 (0.93-1.13) | RERI (95% CI): 0.11 (-0.06-0.27)

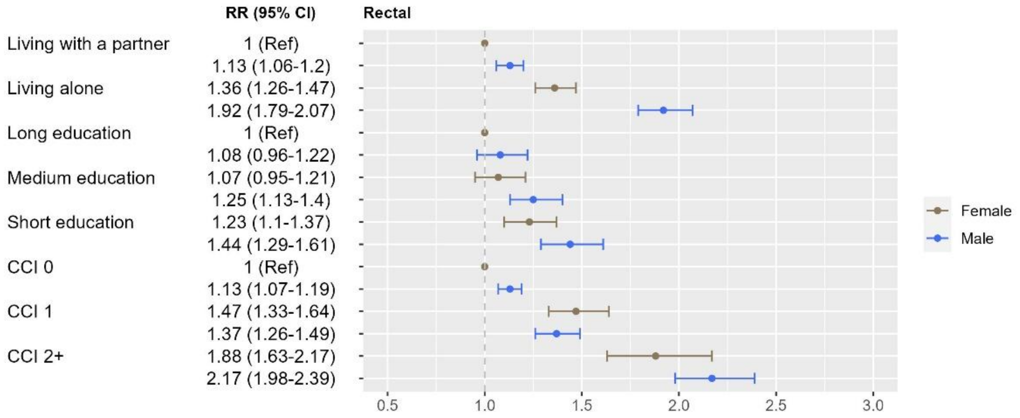

Measure of effect modification on multiplicative scale (RR) and additive scale (RERI):
Cohabitation status: RR (95% CI): 1.26 (1.14-1.38) | RERI (95% CI): 0.44 (0.31-0.56)
Medium education: RR (95% CI): 1.08 (0.94-1.25) | RERI (95% CI): 0.1 (-0.05-0.25)
Short education: RR (95% CI): 1.09 (0.95-1.25) | RERI (95% CI): 0.13 (-0.01-0.28)
CCI 1: RR (95% CI): 0.82 (0.72-0.94) | RERI (95% CI): -0.23 (-0.42--0.05)
CCI 2+: RR (95% CI): 1.02 (0.86-1.21) | RERI (95% CI): 0.16 (-0.16-0.47)

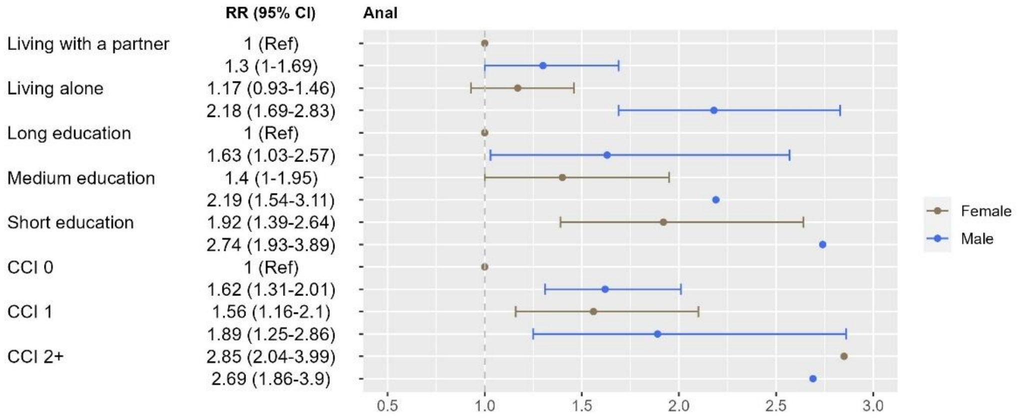

Measure of effect modification on multiplicative scale (RR) and additive scale (RERI):
Cohabitation status: RR (95% CI): 1.44 (1-2.07) | RERI (95% CI): 0.71 (0.18-1.25)
Medium education: RR (95% CI): 0.96 (0.56-1.65) | RERI (95% CI): 0.16 (-0.67-1)
Short education: RR (95% CI): 0.88 (0.52-1.49) | RERI (95% CI): 0.19 (-0.72-1.11)
CCI 1: RR (95% CI): 0.75 (0.44-1.25) | RERI (95% CI): -0.3 (-1.2-0.61)
CCI 2+: RR (95% CI): 0.58 (0.35-0.97) | RERI (95% CI): -0.79 (-2.12-0.55)

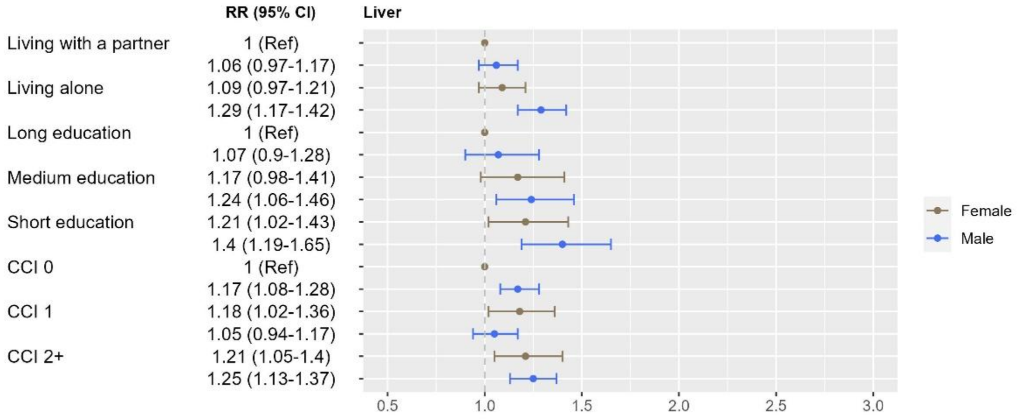

Measure of effect modification on multiplicative scale (RR) and additive scale (RERI):
Cohabitation status: RR (95% CI): 1.11 (0.97-1.28) | RERI (95% CI): 0.14 (0-0.28)
Medium education: RR (95% CI): 0.99 (0.8-1.22) | RERI (95% CI): 0 (-0.22-0.22)
Short education: RR (95% CI): 1.09 (0.89-1.33) | RERI (95% CI): 0.12 (-0.08-0.33)
CCI 1: RR (95% CI): 0.76 (0.64-0.9) | RERI (95% CI): -0.31 (-0.51--0.1)
CCI 2+: RR (95% CI): 0.87 (0.74-1.03) | RERI (95% CI): -0.14 (-0.34-0.06)

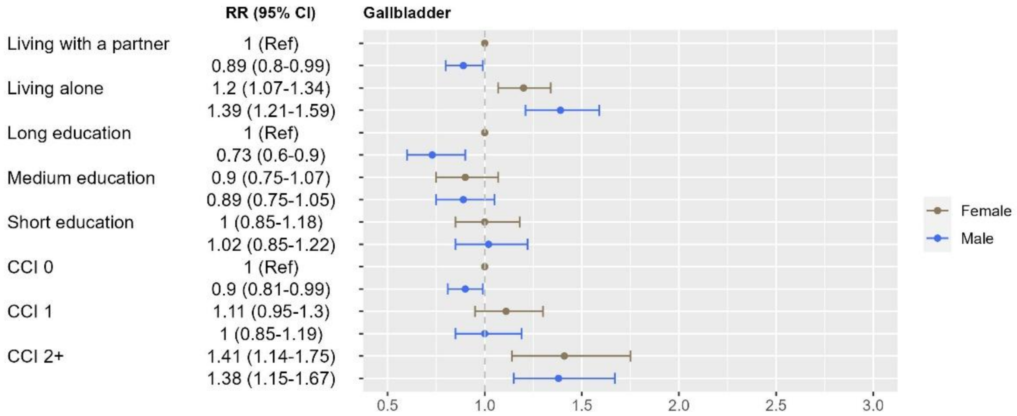

Measure of effect modification on multiplicative scale (RR) and additive scale (RERI):
Cohabitation status: RR (95% CI): 1.3 (1.09-1.55) | RERI (95% CI): 0.3 (0.09-0.5)
Medium education: RR (95% CI): 1.34 (1.05-1.72) | RERI (95% CI): 0.25 (0.06-0.45)
Short education: RR (95% CI): 1.39 (1.09-1.77) | RERI (95% CI): 0.29 (0.09-0.48)
CCI 1: RR (95% CI): 1.01 (0.8-1.27) | RERI (95% CI): 0 (-0.24-0.24)
CCI 2+: RR (95% CI): 1.09 (0.82-1.45) | RERI (95% CI): 0.07 (-0.32-0.46)

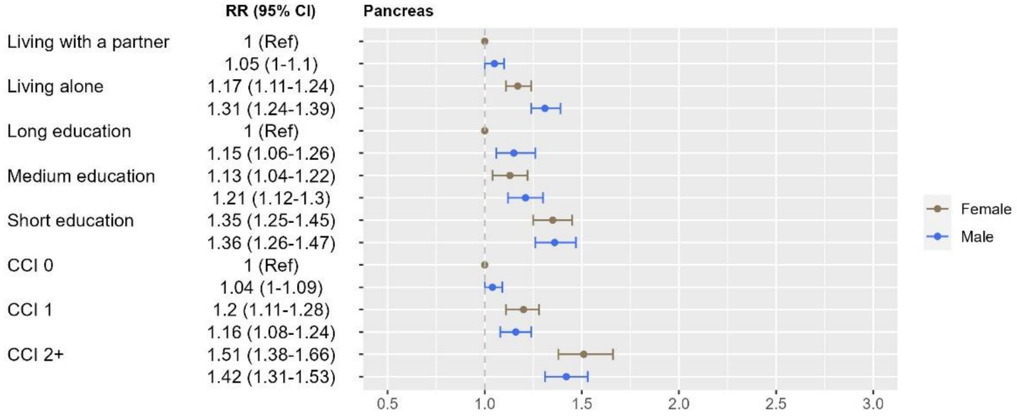

Measure of effect modification on multiplicative scale (RR) and additive scale (RERI):
Cohabitation status: RR (95% CI): 1.07 (0.99-1.15) | RERI (95% CI): 0.09 (0-0.18)
Medium education: RR (95% CI): 0.93 (0.83-1.03) | RERI (95% CI): -0.08 (-0.19-0.04)
Short education: RR (95% CI): 0.87 (0.79-0.97) | RERI (95% CI): -0.14 (-0.27--0.02)
CCI 1: RR (95% CI): 0.93 (0.84-1.02) | RERI (95% CI): -0.08 (-0.2-0.03)
CCI 2+: RR (95% CI): 0.9 (0.8-1.02) | RERI (95% CI): -0.14 (-0.31-0.04)

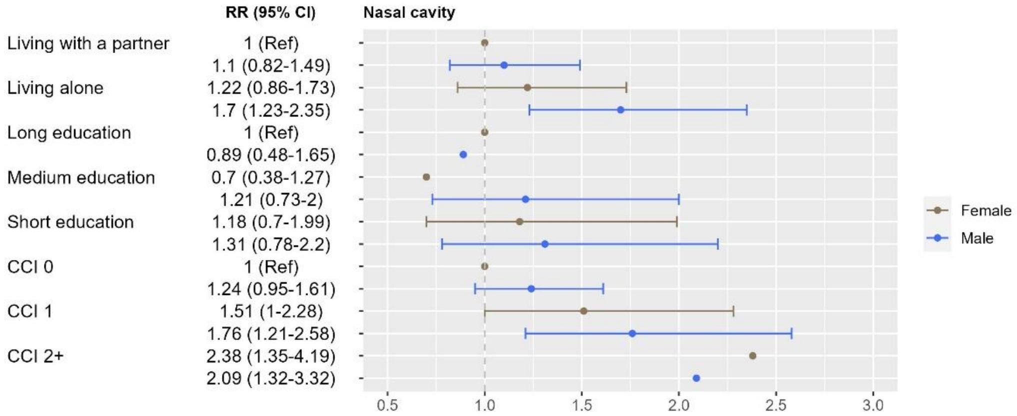

Measure of effect modification on multiplicative scale (RR) and additive scale (RERI):
Cohabitation status: RR (95% CI): 1.27 (0.81-1.97) | RERI (95% CI): 0.38 (-0.14-0.9)
Medium education: RR (95% CI): 1.94 (0.92-4.09) | RERI (95% CI): 0.62 (0.08-1.15)
Short education: RR (95% CI): 1.25 (0.63-2.48) | RERI (95% CI): 0.24 (-0.39-0.87)
CCI 1: RR (95% CI): 0.94 (0.55-1.61) | RERI (95% CI): 0.01 (-0.8-0.83)
CCI 2+: RR (95% CI): 0.71 (0.35-1.45) | RERI (95% CI): -0.52 (-2.08-1.03)

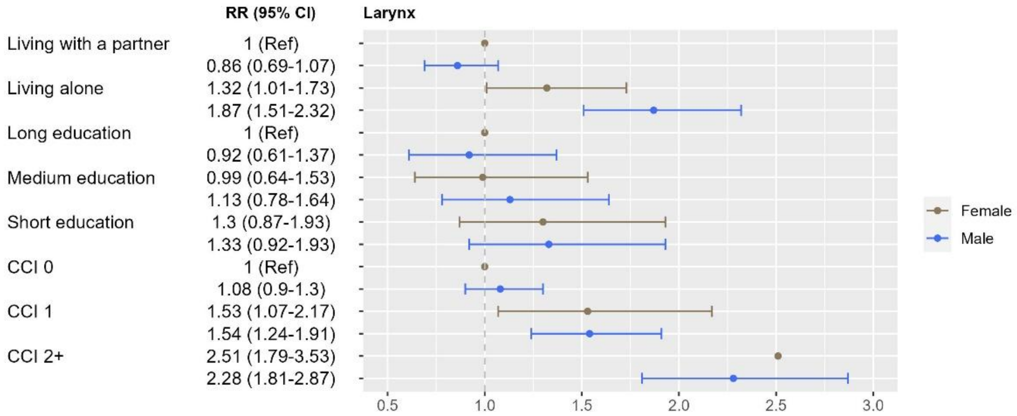

Measure of effect modification on multiplicative scale (RR) and additive scale (RERI):
Cohabitation status: RR (95% CI): 1.64 (1.22-2.19) | RERI (95% CI): 0.68 (0.39-0.98)
Medium education: RR (95% CI): 1.25 (0.77-2.03) | RERI (95% CI): 0.23 (-0.19-0.65)
Short education: RR (95% CI): 1.12 (0.72-1.76) | RERI (95% CI): 0.12 (-0.32-0.56)
CCI 1: RR (95% CI): 0.93 (0.63-1.37) | RERI (95% CI): -0.07 (-0.62-0.48)
CCI 2+: RR (95% CI): 0.84 (0.57-1.23) | RERI (95% CI): -0.31 (-1.17-0.54)

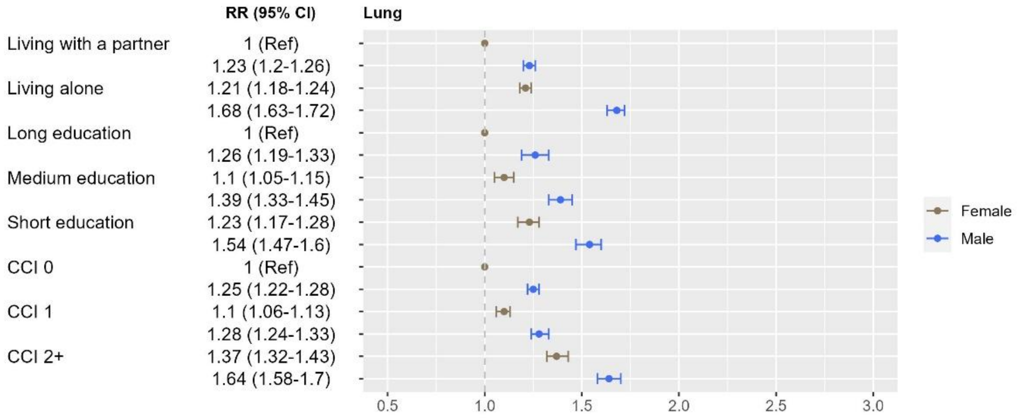

Measure of effect modification on multiplicative scale (RR) and additive scale (RERI):
Cohabitation status: RR (95% CI): 1.13 (1.08-1.17) | RERI (95% CI): 0.23 (0.19-0.28)
Medium education: RR (95% CI): 1 (0.94-1.07) | RERI (95% CI): 0.03 (-0.04-0.1)
Short education: RR (95% CI): 1 (0.94-1.06) | RERI (95% CI): 0.05 (-0.02-0.12)
CCI 1: RR (95% CI): 0.94 (0.9-0.98) | RERI (95% CI): -0.06 (-0.11--0.01)
CCI 2+: RR (95% CI): 0.96 (0.91-1.01) | RERI (95% CI): 0.02 (-0.06-0.1)

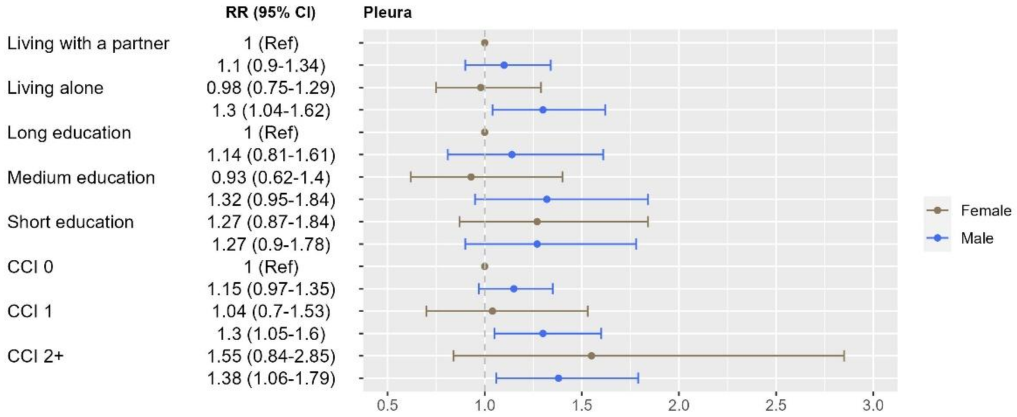

Measure of effect modification on multiplicative scale (RR) and additive scale (RERI):
Cohabitation status: RR (95% CI): 1.2 (0.89-1.63) | RERI (95% CI): 0.22 (-0.08-0.51)
Medium education: RR (95% CI): 1.25 (0.81-1.92) | RERI (95% CI): 0.25 (-0.13-0.63)
Short education: RR (95% CI): 0.88 (0.58-1.32) | RERI (95% CI): -0.14 (-0.62-0.34)
CCI 1: RR (95% CI): 1.09 (0.71-1.65) | RERI (95% CI): 0.11 (-0.34-0.55)
CCI 2+: RR (95% CI): 0.78 (0.41-1.49) | RERI (95% CI): -0.31 (-1.3-0.67)

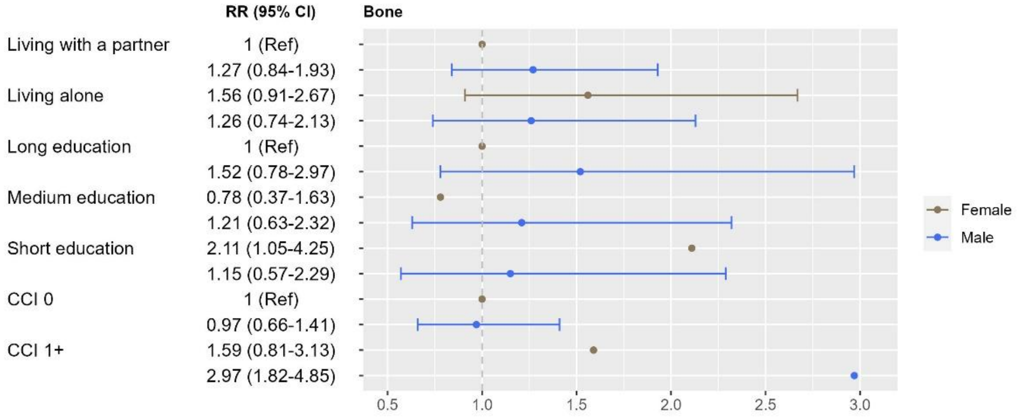

Measure of effect modification on multiplicative scale (RR) and additive scale (RERI):
Cohabitation status: RR (95% CI): 0.63 (0.31-1.28) | RERI (95% CI): -0.58 (-1.59-0.44)
Medium education: RR (95% CI): 1.02 (0.43-2.43) | RERI (95% CI): -0.09 (-1.06-0.88)
Short education: RR (95% CI): 0.36 (0.15-0.84) | RERI (95% CI): -1.48 (-3.25-0.29)
CCI 1+: RR (95% CI): 1.93 (0.87-4.3) | RERI (95% CI): 1.42 (-0.07-2.9)

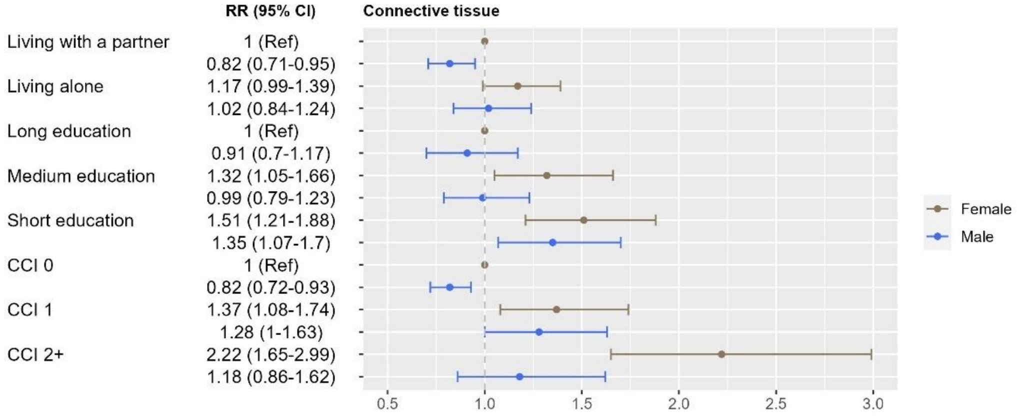

Measure of effect modification on multiplicative scale (RR) and additive scale (RERI):
Cohabitation status: RR (95% CI): 1.06 (0.83-1.36) | RERI (95% CI): 0.03 (-0.22-0.28)
Medium education: RR (95% CI): 0.82 (0.6-1.13) | RERI (95% CI): -0.24 (-0.59-0.11)
Short education: RR (95% CI): 0.99 (0.72-1.35) | RERI (95% CI): -0.06 (-0.42-0.3)
CCI 1: RR (95% CI): 1.14 (0.81-1.61) | RERI (95% CI): 0.09 (-0.34-0.52)
CCI 2+: RR (95% CI): 0.65 (0.42-1) | RERI (95% CI): -0.86 (-1.61--0.12)

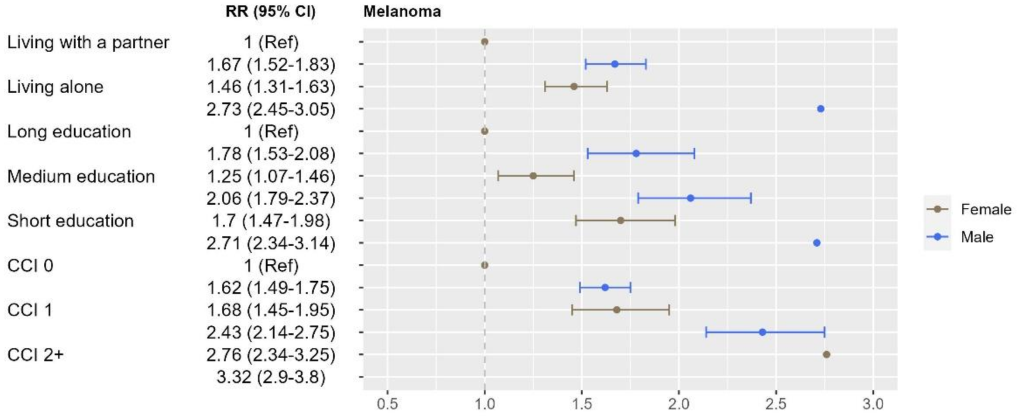

Measure of effect modification on multiplicative scale (RR) and additive scale (RERI):
Cohabitation status: RR (95% CI): 1.12 (0.97-1.29) | RERI (95% CI): 0.6 (0.35-0.85)
Medium education: RR (95% CI): 0.92 (0.76-1.12) | RERI (95% CI): 0.03 (-0.24-0.29)
Short education: RR (95% CI): 0.89 (0.74-1.08) | RERI (95% CI): 0.23 (-0.07-0.53)
CCI 1: RR (95% CI): 0.89 (0.74-1.08) | RERI (95% CI): 0.13 (-0.23-0.48)
CCI 2+: RR (95% CI): 0.74 (0.6-0.91) | RERI (95% CI): -0.06 (-0.64-0.52)

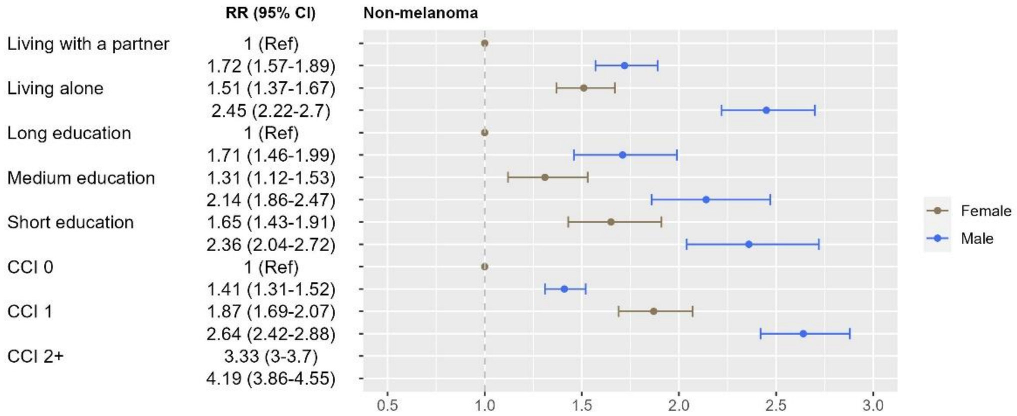

Measure of effect modification on multiplicative scale (RR) and additive scale (RERI):
Cohabitation status: RR (95% CI): 0.94 (0.84-1.05) | RERI (95% CI): 0.21 (0.04-0.39)
Medium education: RR (95% CI): 0.96 (0.8-1.15) | RERI (95% CI): 0.13 (-0.1-0.36)
Short education: RR (95% CI): 0.84 (0.71-0.99) | RERI (95% CI): 0 (-0.24-0.23)
CCI 1: RR (95% CI): 1 (0.88-1.14) | RERI (95% CI): 0.37 (0.12-0.61)
CCI 2+: RR (95% CI): 0.89 (0.79-1.02) | RERI (95% CI): 0.45 (0.07-0.84)

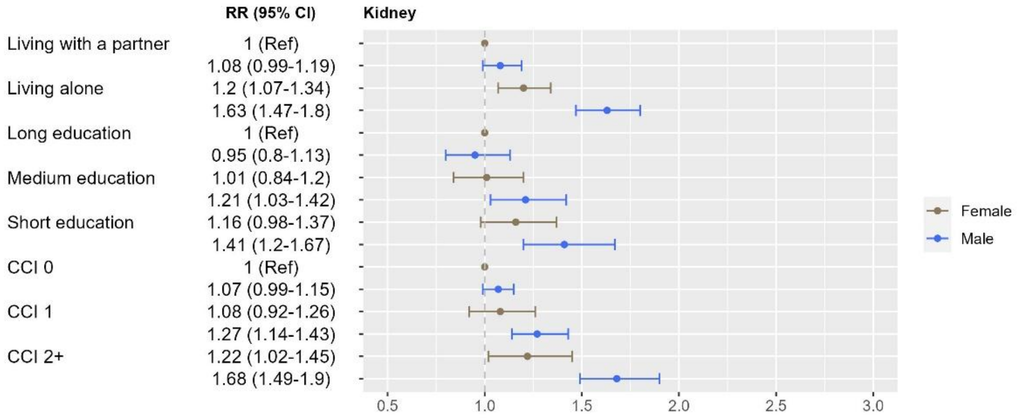

Measure of effect modification on multiplicative scale (RR) and additive scale (RERI):
Cohabitation status: RR (95% CI): 1.25 (1.09-1.44) | RERI (95% CI): 0.35 (0.18-0.51)
Medium education: RR (95% CI): 1.27 (1.03-1.56) | RERI (95% CI): 0.25 (0.07-0.44)
Short education: RR (95% CI): 1.28 (1.05-1.57) | RERI (95% CI): 0.3 (0.12-0.49)
CCI 1: RR (95% CI): 1.11 (0.92-1.34) | RERI (95% CI): 0.13 (-0.08-0.34)
CCI 2+: RR (95% CI): 1.29 (1.05-1.59) | RERI (95% CI): 0.39 (0.12-0.67)

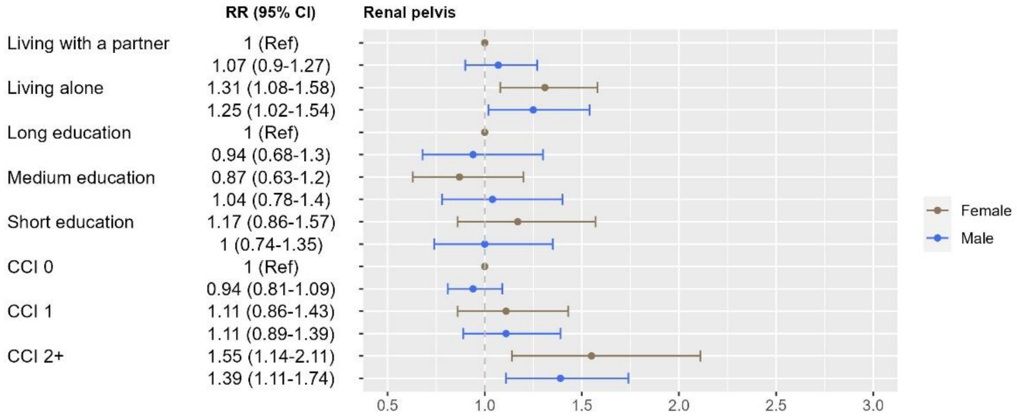

Measure of effect modification on multiplicative scale (RR) and additive scale (RERI):
Cohabitation status: RR (95% CI): 0.9 (0.69-1.16) | RERI (95% CI): -0.12 (-0.43-0.19)
Medium education: RR (95% CI): 1.28 (0.87-1.89) | RERI (95% CI): 0.24 (-0.1-0.57)
Short education: RR (95% CI): 0.91 (0.63-1.33) | RERI (95% CI): -0.11 (-0.5-0.29)
CCI 1: RR (95% CI): 1.07 (0.76-1.48) | RERI (95% CI): 0.06 (-0.3-0.42)
CCI 2+: RR (95% CI): 0.96 (0.66-1.39) | RERI (95% CI): -0.1 (-0.64-0.45)

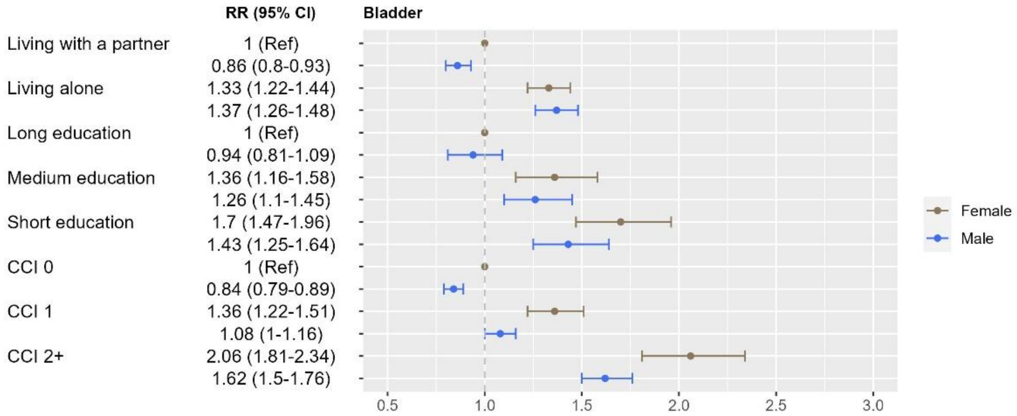

Measure of effect modification on multiplicative scale (RR) and additive scale (RERI):
Cohabitation status: RR (95% CI): 1.19 (1.08-1.32) | RERI (95% CI): 0.18 (0.07-0.29)
Medium education: RR (95% CI): 0.99 (0.83-1.18) | RERI (95% CI): -0.03 (-0.23-0.16)
Short education: RR (95% CI): 0.9 (0.76-1.05) | RERI (95% CI): -0.21 (-0.41-0)
CCI 1: RR (95% CI): 0.94 (0.83-1.07) | RERI (95% CI): -0.12 (-0.28-0.03)
CCI 2+: RR (95% CI): 0.94 (0.81-1.08) | RERI (95% CI): -0.27 (-0.54--0.01)

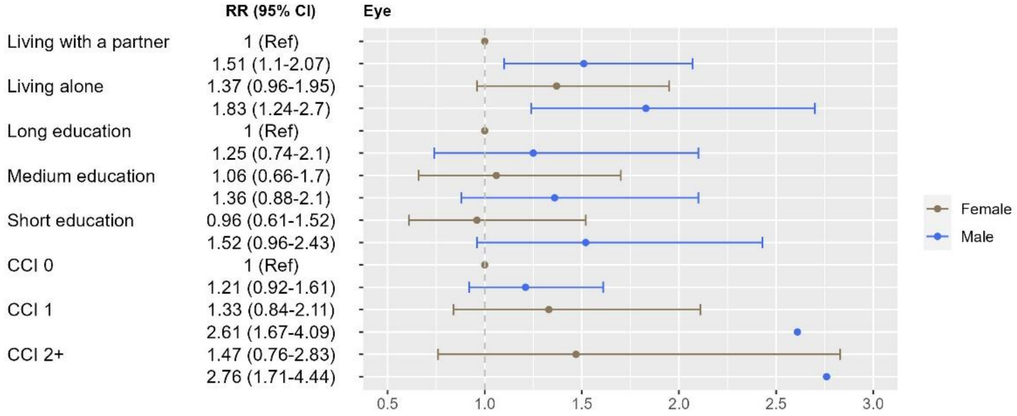

Measure of effect modification on multiplicative scale (RR) and additive scale (RERI):
Cohabitation status: RR (95% CI): 0.89 (0.54-1.46) | RERI (95% CI): -0.05 (-0.8-0.7)
Medium education: RR (95% CI): 1.03 (0.54-1.97) | RERI (95% CI): 0.05 (-0.68-0.79)
Short education: RR (95% CI): 1.27 (0.66-2.43) | RERI (95% CI): 0.31 (-0.41-1.04)
CCI 1: RR (95% CI): 1.61 (0.85-3.05) | RERI (95% CI): 1.07 (-0.14-2.27)
CCI 2+: RR (95% CI): 1.55 (0.69-3.46) | RERI (95% CI): 1.07 (-0.44-2.59)

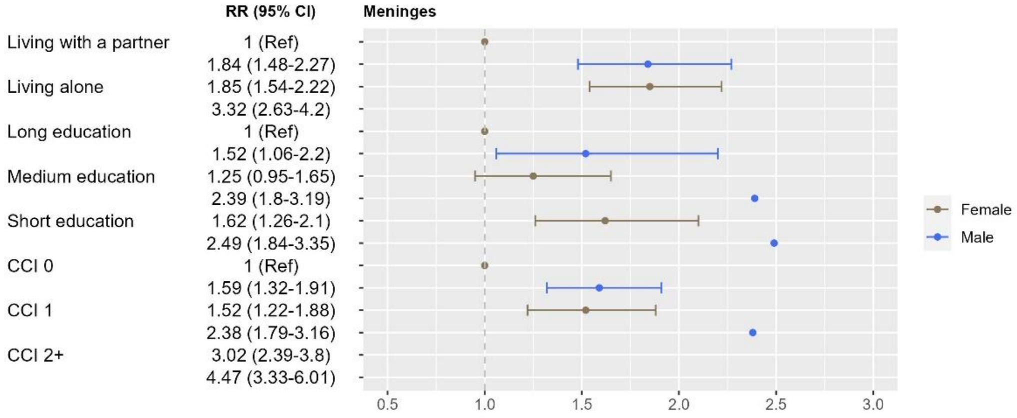

Measure of effect modification on multiplicative scale (RR) and additive scale (RERI):
Cohabitation status: RR (95% CI): 0.98 (0.72-1.32) | RERI (95% CI): 0.63 (-0.06-1.33)
Medium education: RR (95% CI): 1.26 (0.81-1.95) | RERI (95% CI): 0.62 (-0.02-1.27)
Short education: RR (95% CI): 1.01 (0.65-1.55) | RERI (95% CI): 0.34 (-0.35-1.03)
CCI 1: RR (95% CI): 0.98 (0.68-1.43) | RERI (95% CI): 0.27 (-0.47-1)
CCI 2+: RR (95% CI): 0.93 (0.63-1.38) | RERI (95% CI): 0.87 (-0.53-2.26)

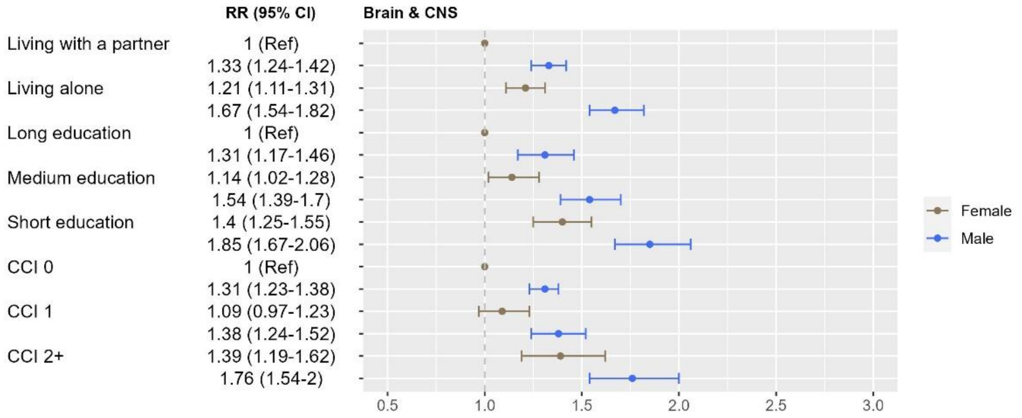

Measure of effect modification on multiplicative scale (RR) and additive scale (RERI):
Cohabitation status: RR (95% CI): 1.04 (0.93-1.17) | RERI (95% CI): 0.14 (-0.01-0.28)
Medium education: RR (95% CI): 1.03 (0.9-1.19) | RERI (95% CI): 0.09 (-0.07-0.25)
Short education: RR (95% CI): 1.02 (0.88-1.17) | RERI (95% CI): 0.15 (-0.03-0.33)
CCI 1: RR (95% CI): 0.97 (0.83-1.13) | RERI (95% CI): -0.02 (-0.21-0.17)
CCI 2+: RR (95% CI): 0.97 (0.79-1.19) | RERI (95% CI): 0.06 (-0.24-0.37)

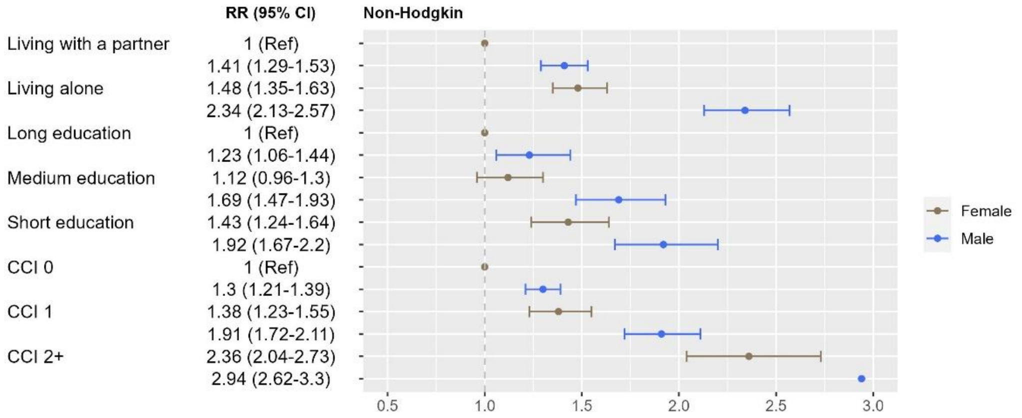

Measure of effect modification on multiplicative scale (RR) and additive scale (RERI):
Cohabitation status: RR (95% CI): 1.12 (1-1.27) | RERI (95% CI): 0.45 (0.27-0.64)
Medium education: RR (95% CI): 1.22 (1.02-1.47) | RERI (95% CI): 0.34 (0.14-0.53)
Short education: RR (95% CI): 1.09 (0.92-1.3) | RERI (95% CI): 0.26 (0.06-0.46)
CCI 1: RR (95% CI): 1.06 (0.91-1.24) | RERI (95% CI): 0.23 (0-0.46)
CCI 2+: RR (95% CI): 0.96 (0.8-1.15) | RERI (95% CI): 0.28 (-0.16-0.72)

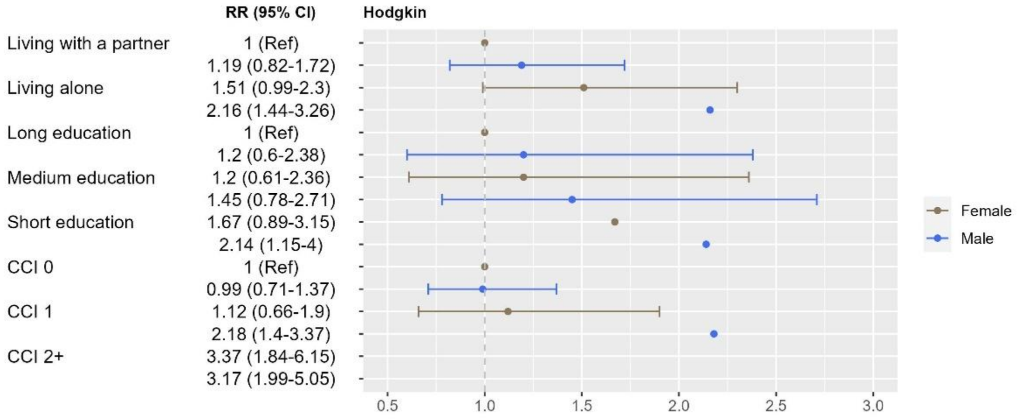

Measure of effect modification on multiplicative scale (RR) and additive scale (RERI):
Cohabitation status: RR (95% CI): 1.2 (0.69-2.08) | RERI (95% CI): 0.46 (-0.35-1.26)
Medium education: RR (95% CI): 1.01 (0.44-2.3) | RERI (95% CI): 0.06 (-0.86-0.97)
Short education: RR (95% CI): 1.07 (0.49-2.35) | RERI (95% CI): 0.28 (-0.68-1.23)
CCI 1: RR (95% CI): 1.98 (1.01-3.86) | RERI (95% CI): 1.07 (0.11-2.03)
CCI 2+: RR (95% CI): 0.95 (0.45-2.02) | RERI (95% CI): -0.18 (-2.43-2.07)

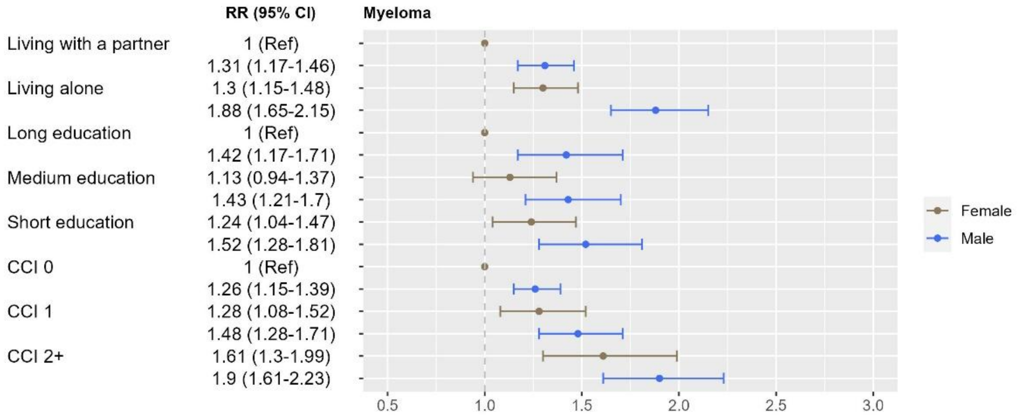

Measure of effect modification on multiplicative scale (RR) and additive scale (RERI):
Cohabitation status: RR (95% CI): 1.1 (0.93-1.31) | RERI (95% CI): 0.27 (0.03-0.51)
Medium education: RR (95% CI): 0.89 (0.71-1.13) | RERI (95% CI): -0.12 (-0.4-0.17)
Short education: RR (95% CI): 0.87 (0.69-1.08) | RERI (95% CI): -0.13 (-0.42-0.15)
CCI 1: RR (95% CI): 0.91 (0.73-1.14) | RERI (95% CI): -0.07 (-0.36-0.23)
CCI 2+: RR (95% CI): 0.93 (0.72-1.22) | RERI (95% CI): 0.03 (-0.41-0.46)

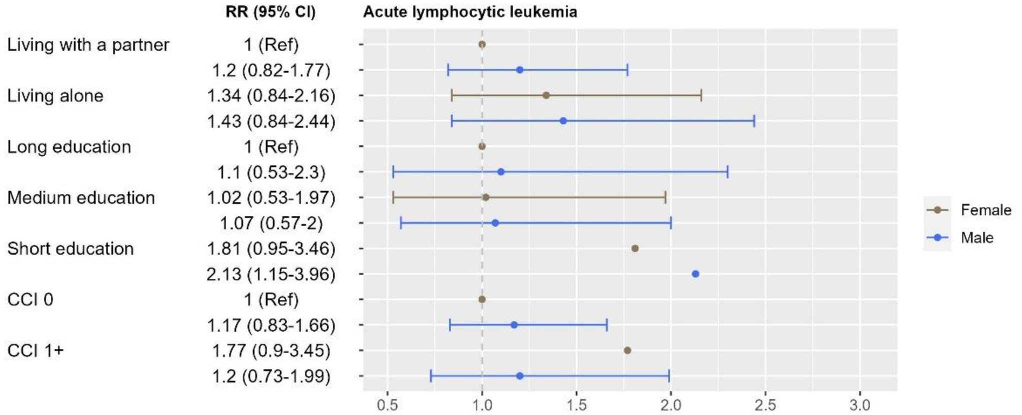

Measure of effect modification on multiplicative scale (RR) and additive scale (RERI):
Cohabitation status: RR (95% CI): 0.88 (0.44-1.77) | RERI (95% CI): -0.12 (-1.04-0.8)
Medium education: RR (95% CI): 0.94 (0.38-2.32) | RERI (95% CI): -0.06 (-1.03-0.9)
Short education: RR (95% CI): 1.06 (0.44-2.55) | RERI (95% CI): 0.21 (-0.94-1.37)
CCI 1+: RR (95% CI): 0.58 (0.25-1.34) | RERI (95% CI): -0.74 (-2.06-0.58)

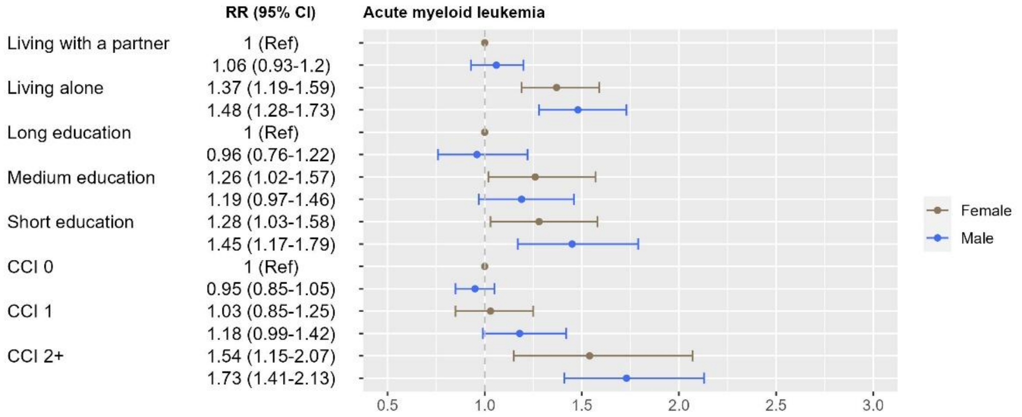

Measure of effect modification on multiplicative scale (RR) and additive scale (RERI):
Cohabitation status: RR (95% CI): 1.02 (0.84-1.24) | RERI (95% CI): 0.05 (-0.19-0.3)
Medium education: RR (95% CI): 0.98 (0.74-1.3) | RERI (95% CI): -0.04 (-0.34-0.27)
Short education: RR (95% CI): 1.18 (0.89-1.55) | RERI (95% CI): 0.21 (-0.08-0.49)
CCI 1: RR (95% CI): 1.21 (0.94-1.57) | RERI (95% CI): 0.21 (-0.07-0.48)
CCI 2+: RR (95% CI): 1.18 (0.83-1.69) | RERI (95% CI): 0.24 (-0.31-0.79)

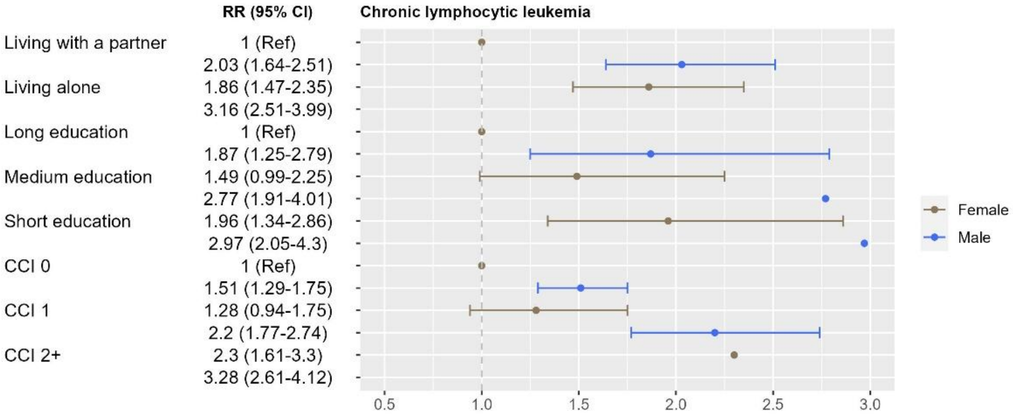

Measure of effect modification on multiplicative scale (RR) and additive scale (RERI):
Cohabitation status: RR (95% CI): 0.84 (0.64-1.11) | RERI (95% CI): 0.28 (-0.24-0.8)
Medium education: RR (95% CI): 1 (0.62-1.59) | RERI (95% CI): 0.41 (-0.2-1.03)
Short education: RR (95% CI): 0.81 (0.52-1.26) | RERI (95% CI): 0.15 (-0.49-0.78)
CCI 1: RR (95% CI): 1.14 (0.79-1.65) | RERI (95% CI): 0.42 (-0.14-0.97)
CCI 2+: RR (95% CI): 0.94 (0.62-1.43) | RERI (95% CI): 0.47 (-0.55-1.48)

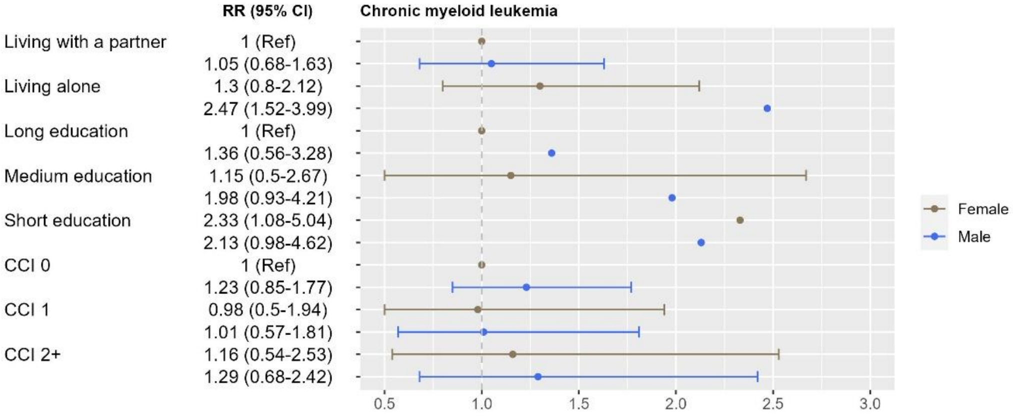

Measure of effect modification on multiplicative scale (RR) and additive scale (RERI):
Cohabitation status: RR (95% CI): 1.8 (0.95-3.42) | RERI (95% CI): 1.11 (0.16-2.06)
Medium education: RR (95% CI): 1.26 (0.44-3.58) | RERI (95% CI): 0.46 (-0.69-1.61)
Short education: RR (95% CI): 0.67 (0.25-1.8) | RERI (95% CI): -0.56 (-2.19-1.07)
CCI 1: RR (95% CI): 0.84 (0.35-2.02) | RERI (95% CI): -0.2 (-1.11-0.71)
CCI 2+: RR (95% CI): 0.9 (0.34-2.38) | RERI (95% CI): -0.11 (-1.28-1.07)

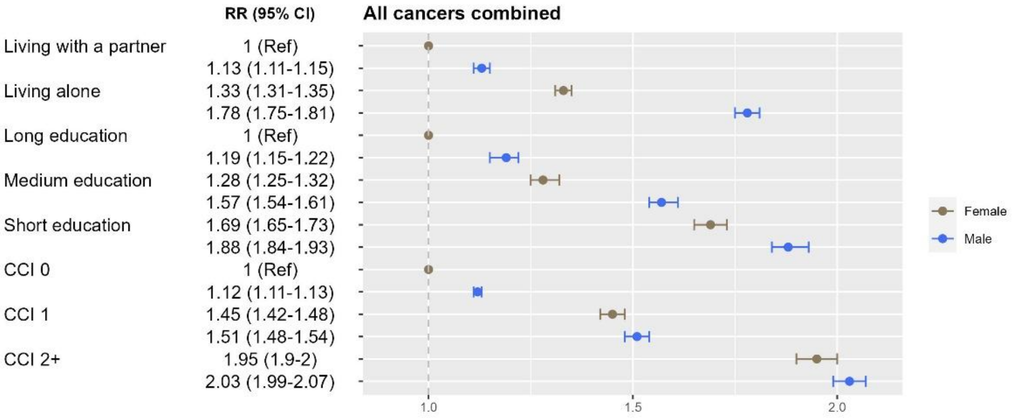

Measure of effect modification on multiplicative scale (RR) and additive scale (RERI):
Cohabitation status: RR (95% CI) = 1.18 (1.16-1.21) | RERI (95% CI) = 0.32 (0.29-0.35)
Medium education: RR (95% CI) = 1.03 (1-1.07) | RERI (95% CI) = 0.1 (0.07-0.14)
Short education: RR (95% CI) = 0.94 (0.91-0.97) | RERI (95% CI) = 0.01 (-0.03-0.05)
CCI 1: RR (95% CI) = 0.93 (0.9-0.95) | RERI (95% CI) = -0.06 (-0.1--0.02)
CCI 2+: RR (95% CI) = 0.93 (0.9-0.96) | RERI (95% CI) = -0.04 (-0.1-0.02)

Figure 3: IRRs by EMRs comparing males with females aged ≥ 30 years at diagnosis, for cancers related to alcohol and/or smoking, stratified by cohabitation status

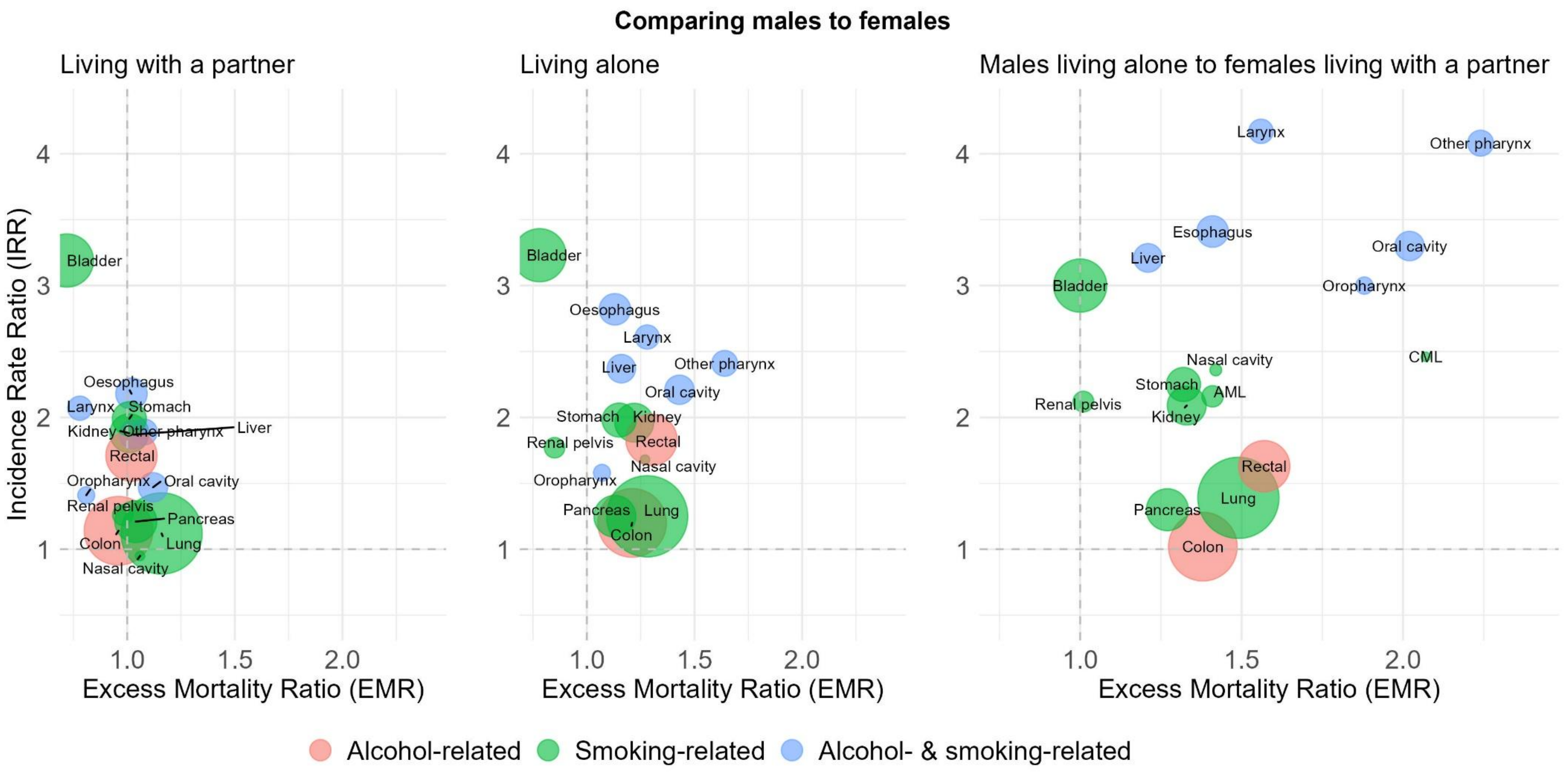

The size of the points corresponds to the cancer burden, with larger points indicating greater cancer burden. All estimates are adjusted for age and year of diagnosis. Acute myeloid leukaemia (AML), Chronic lymphocytic leukaemia (CML).

Table 1: Classification of cancer sites

Cancer sites	ICD-10 codes
**Head and neck**	
Oral cavity	C00-C06
Salivary glands	C07-C08
Oropharynx	C09-C10
Other pharynx	C11-C14
Thyroid	C73
**Digestive organs**	
Oesophagus	C15
Stomach	C16
Small intestine	C17
Colon	C18-C19
Rectal	C20
Anal	C21
Liver	C22
Gallbladder	C23-C24
Pancreas	C25
**Respiratory system and intrathoracic organs**	
Nasal cavities	C30-C31
Larynx	C32
Lung and trachea	C33-C34
Pleura	C384, C450
**Bones and connective tissue**	
Bone and cartilage	C40-C41
Connective tissue	C47, C48-C49
**Skin**	
Malignant melanoma	C43
Non-melanoma[a]	C44
**Urinary tract**	
Kidney	C64
Renal pelvis[b]	C65-C66, C68
	D301 - D302; D41 1-D413, D417, D418
	D091; D304-D309
	D417-D419
Bladder[b]	C67
	D090; D303; D414

**Central nervous system**	
Eye	C69
Meninges	C70
	D32; D42
Brain and CNS	C71-C72; C751-C753
	D330-D332; D430-D432
	D352-D354, D443-D445
	D333-D339; D433-D439
**Hematological**	**C81-C96**
Hodgkin lymphoma	C81
Non-Hodgkin lymphoma	C82-C86; C884
Multiple myeloma	C90
Acute lymphocytic leukaemia	**ICD-O morfo 3:** 9826, 9835-9837
Chronic lymphocytic leukaemia	**ICD-O morfo 3:** 9823
Acute myeloid leukaemia	**ICD-O morfo 3:** 9840, 9861, 9866, 9867, 9870-9874, 9891, 9895-9897, 9910, 9920
Chronic myeloid leukaemia	**ICD-O morfo 3:** 9863, 9875, 9876, 9946
**All cancers combined**	Any of the above codes

[a] Excluding basal cell carcinoma, morphology code=809X3.

[b] D-codes are restricted to morphology codes 812X-813X.

Table 2: Categorisation of cancer sites into etiological groups based on classifications from the International Agency for Research on Cancer (IARC)

Exposure	Cancer	ICD-10 codes
**Alcohol-related cancers (1)**		
	Oral cavity	C00-C06
	Oropharynx	C09-C10
	Other pharynx	C11-C14
	Oesophagus	C15
	Liver	C22
	Colon	C18-C19
	Rectal	C20
	Larynx	C32
**Smoking-related cancers (1)**	Liver	C22
	Oral cavity	C00-C06
	Oropharynx	C10
	Other pharynx	C09, C11-C14
	Oesophagus	C15
	Stomach	C16
	Pancreas	C25
	Nasal cavity	C30-C31
	Larynx	C32
	Lung, bronchus and trachea	C33-C34
	Kidney	C64
	Renal pelvis	C65-C66, C68
	Bladder	C67 (D090; D303; D414) D-codes are restricted to morphology codes 812X-813X.
	Acute myeloid leukaemia	**ICD-O morfo 3:** 9840, 9861, 9866, 9867, 9870-9874, 9891, 9895-9897, 9910, 9920
	Chronic myeloid leukaemia	**ICD-O morfo 3:** 9863, 9875, 9876, 9946
**Diet-related cancers (2)**<br>Processed and red meat	Colon	C18-C19
	Rectal	C20
**Virus-related cancers (3)**		
HPV 16	Oral cavity	C00-C06
HPV 16 (Tonsils)<br>*Ebstein Bar (Nasopharynx)*	Other pharynx	C09, C11-C14
Helicobacter pylori	Stomach	C16
HPV 16	Oropharynx	C10
Hepatitis B (Hepatocellular carcinoma)<br>*Hepatitis C (Hepatocellular carcinoma)*<br>*Opisthorchis viverrine & Clonorchis sinensis (Cholangiocarcinoma)*	Liver	C22
HPV 16<br>*HIV-1*	Anal	C21

Epstein Bar<br>*Hepatitis C*<br>*HIV-1*<br>*Human T-cell lymphotropic virus type 1*	Non-Hodgkin lymphoma	C82-C86, C884
Epstein Bar<br>*HIV-1*	Hodgkin lymphoma	C81

References

1. International Agency for Research on Cancer (IARC). Personal Habits and Indoor Combustions - A Review of Human Carcinogens. Lyon, France: Working Group on the Evaluation of Carcinogenic Risks to Humans; 2012.

2. International Agency for Research on Cancer (IARC). Red Meat and Processed Meat. Lyon, France: Working Group on the Evaluation of Carcinogenic Risks to Humans; 2018.

3. International Agency for Research on Cancer (IARC). Biological Agents - A review of Human Carcinogens. Lyon, France: Working Group on the Evaluation of Carcinogenic Risks to Humans; 2000.

Table 3: Individuals included in the effect modification analyses

		**Males**<br>**n = 197,375**	**Females**<br>**n = 157,964**
**Excluded**			
	< 30 years at diagnosis	n = 3,723	n = 4,391
	Born in or before 1920	n = 3,850	n = 5,403
	Immigration < 5 years prior to diagnosis	n = 1,322	n = 985
	Missing values on cohabitation	n = 347	n = 147
	Missing values on education	n = 3,892	n = 2,230
		**n = 184,241**<br>*Cancers =184,635*[a]	**n = 144,808**<br>*Cancers = 145,010*[a]

[a] Some individuals had more than one first primary cancer registered on the same date of diagnosis.

Table 4: Number of cancer cases (n), proportions (%), number of events, person-years, and mean age at diagnosis for 35 non-sex-specific cancer sites, for males and females

		Males				Females			
Cancer site		n (%)[a]	Events[b] (%)	Person-years[c]	Mean age (SD)[d]	n (%)[a]	Events[b] (%)	Person-years[c]	Mean age (SD)[d]
**Head and neck**	Oral cavity	3,640 (2)	1,580 (2)	22,747,120	63 (11)	2,034 (1)	791 (1)	20,732,428	67 (13)
	Salivary glands	433 (0)	165 (0)	9,258,408	64 (16)	395 (0)	90 (0)	10,155,090	60 (19)
	Oropharynx	2,959 (1)	1,060 (1)	20,269,518	61 (9)	1,025 (1)	358 (1)	15,631,032	61 (10)
	Other pharynx	1,338 (1)	833 (1)	17,040,433	62 (11)	336 (0)	336 (0)	8,422,885	61 (13)
	Thyroid	1,179 (1)	186 (0)	22,241,644	54 (16)	3,077 (2)	292 (0)	33,411,955	51 (17)
**Digestive organs**	Oesophagus	4,960 (3)	4,037 (4)	20,075,051	68 (10)	1,791 (1)	1,442 (2)	16,512,515	70 (11)
	Stomach	5,509 (3)	4,090 (4)	23,366,307	68 (12)	2,743 (2)	2,006 (3)	21,901,676	69 (13)
	Small intestine	1,009 (1)	450 (0)	13,861,938	66 (12)	900 (1)	382 (1)	13,957,948	66 (13)
	Colon	21,515 (11)	9,242 (10)	29,534,487	70 (11)	21,534 (14)	9,220 (13)	31,051,425	71 (12)
	Rectal	13,265 (7)	5,229 (6)	25,688,467	68 (11)	8,444 (5)	3,190 (5)	26,393,272	69 (13)
	Anal	561 (0)	211 (0)	11,210,180	63 (12)	1,308 (1)	357 (1)	18,411,869	63 (13)
	Liver	3,764 (2)	3,259 (3)	20,680,254	67 (12)	1,609 (1)	1,361 (2)	17,931,488	69 (15)
	Gallbladder	1,220 (1)	1,004 (1)	14,279,318	68 (12)	1,605 (1)	1,369 (2)	15,675,299	71 (12)
	Pancreas	6,905 (3)	6,139 (6)	22,365,706	69 (11)	6,636 (4)	5,934 (9)	23,149,429	71 (12)
**Respiratory**	Nasal cavity	544 (0)	241 (0)	10,587,325	65 (14)	378 (0)	152 (0)	8,286,665	65 (16)
	Larynx	2,891 (1)	1,135 (1)	19,115,000	66 (10)	622 (0)	236 (0)	11,354,429	65 (11)
	Lung and trachea	31,256 (16)	25,819 (27)	25,502,398	69 (10)	30,141 (19)	23,046 (34)	27,334,327	69 (10)
	Pleura	1,425 (1)	1,222 (1)	12,653,209	70 (9)	263 (0)	223 (0)	5,579,473	70 (12)
**Bone and connective tissue**	Bone and cartilage	502 (0)	155 (0)	14,306,247	44 (23)	363 (0)	85 (0)	10,823,709	41 (23)
	Connective tissue	1,673 (1)	641 (1)	25,031,957	59 (19)	1,567 (1)	690 (1)	25,151,345	60 (20)
**Skin**	Malignant melanoma	14,306 (7)	2,315 (2)	37,084,342	61 (15)	16,903 (11)	1,656 (2)	39,867,735	56 (17)
	Non-melanoma	18,252 (9)	4,792 (5)	25,566,176	75 (11)	14,243 (9)	3,225 (5)	27,630,269	75 (13)
**Urinary tract**	Kidney	7,387 (4)	2,696 (3)	27,875,857	64 (13)	3,825 (2)	1,435 (2)	25,918,425	66 (14)
	Renal pelvis	1,423 (1)	659 (1)	14,286,564	69 (11)	935 (1)	471 (1)	11,699,532	72 (11)
	Bladder	17,901 (9)	6,705 (7)	27,036,197	70 (11)	5,949 (4)	2,477 (4)	22,879,001	70 (12)

**Central nervous system**	Eye	669 (0)	162 (0)	13,988,882	57 (21)	669 (0)	140 (0)	13,841,120	59 (21)
	Meninges	1,883 (1)	301 (0)	23,200,793	62 (15)	5,439 (3)	613 (1)	30,153,201	62 (15)
	Brain and CNS	9,260 (5)	3,712 (4)	46,528,263	55 (19)	8,358 (5)	2,715 (4)	46,259,139	54 (20)
**Hematological**	Hodgkin lymphoma	1,216 (1)	159 (0)	27,011,069	44 (20)	914 (1)	115 (0)	21,818,930	41 (21)
	Non-Hodgkin lymphoma	9,557 (5)	3,017 (3)	36,429,331	65 (15)	7,439 (5)	2,108 (3)	32,348,363	67 (15)
	Multiple myeloma	3,405 (2)	1,527 (2)	20,066,757	69 (11)	2,596 (2)	1,096 (2)	19,640,829	70 (12)
	Acute lymphocytic leukaemia	605 (0)	128 (0)	14,692,395	22 (23)	437 (0)	113 (0)	10,485,178	23 (26)
	Acute myeloid leukaemia	1,519 (1)	1,100 (1)	20,829,641	65 (18)	1,223 (1)	839 (1)	20,242,555	63 (20)
	Chronic lymphocytic leukaemia	3,364 (2)	763 (1)	19,403,197	68 (11)	2,100 (1)	410 (1)	17,664,430	70 (11)
	Chronic myeloid leukaemia	502 (0)	104 (0)	12,816,927	55 (18)	382 (0)	75 (0)	9,885,227	58 (17)
**All cancers combined**[e]		197,375 (56)	94,581 (58)	49,876,227	67 (14)	157,964 (44)	68,770 (42)	49,956,721	66 (16)

[a] Number and proportion (%) of cancer cases in the cohort diagnosed in 2004-2020.

[b] Number and proportion (%) of deaths up to five years from date of diagnosis.

[c] Number of person-years. The study cohort was followed from January 1, 2004, immigration, or date of birth, whichever occurred last, until date of cancer diagnosis, emigration, age 100 years, death or end of study period on December 31, 2020, whichever occurred first.

[d] Standard deviation.

[e] Defined as any of the 35 examined non-sex-specific cancer sites.

Table 5: Excess mortality ratios (EMRs) with 95% confidence intervals (CI) comparing males with females for 35 non-sex-specific cancer sites, by age groups 18-64 and 65-99, respectively

		**18-64 years**	**65-99 years**
**Cancer site**		**EMR[a] (95% CI)[b]**	**EMR[a] (95% CI)[b]**
**Head and neck**	Oral cavity	1.45 (1.26-1.68)	1.15 (1.00-1.32)
	Salivary glands	1.79 (1.11-2.88)	2.08 (1.35-3.22)
	Oropharynx	0.97 (0.76-1.24)	0.86 (0.64-1.15)
	Other pharynx	1.45 (1.22-1.72)	1.19 (1.00-1.43)
	Thyroid	2.49 (1.67-3.70)	1.58 (1.19-2.11)
**Digestive organs**	Oesophagus	1.13 (1.01-1.26)	1.01 (0.94-1.09)
	Stomach	1.09 (0.99-1.20)	1.01 (0.95-1.09)
	Small intestine	1.07 (0.82-1.40)	1.09 (0.91-1.31)
	Colon	1.04 (0.98-1.12)	1.05 (1.01-1.09)
	Rectal	1.22 (1.10-1.34)	1.07 (1.00-1.14)
	Anal	1.97 (1.49-2.61)	1.27 (0.96-1.68)
	Liver	1.20 (1.06-1.35)	0.99 (0.91-1.07)
	Gallbladder	0.93 (0.79-1.09)	0.92 (0.83-1.02)
	Pancreas	1.15 (1.08-1.24)	1.03 (0.99-1.08)
**Respiratory**	Nasal cavity	1.08 (0.74-1.57)	1.29 (0.95-1.76)
	Larynx	1.07 (0.84-1.35)	0.89 (0.72-1.10)
	Lung and trachea	1.28 (1.24-1.32)	1.14 (1.12-1.17)
	Pleura	1.10 (0.84-1.43)	1.21 (1.01-1.45)
**Bone and connective tissue**	Bone and cartilage	1.76 (1.16-2.67)	0.73 (0.44-1.23)
	Connective tissue	0.96 (0.80-1.14)	0.67 (0.57-0.80)
**Skin**	Malignant melanoma	1.98 (1.71-2.30)	1.75 (1.50-2.06)
	Non-melanoma	2.86 (1.87-4.37)	1.79 (1.48-2.17)
**Urinary tract**	Kidney	1.15 (1.02-1.29)	1.05 (0.95-1.15)
	Renal pelvis	0.92 (0.69-1.24)	0.91 (0.77-1.06)
	Bladder	0.77 (0.68-0.87)	0.72 (0.67-0.77)
**Central nervous system**	Eye	0.79 (0.53-1.18)	2.36 (1.48-3.77)
	Meninges	2.42 (1.58-3.70)	1.53 (1.16-2.02)
	Brain	1.45 (1.34-1.58)	1.14 (1.06-1.23)
**Hematological**	Hodgkin lymphoma	1.21 (0.77-1.91)	0.97 (0.67-1.40)
	Non-Hodgkin lymphoma	1.44 (1.26-1.65)	1.19 (1.10-1.29)
	Multiple myeloma	1.12 (0.93-1.35)	1.25 (1.13-1.39)
	Acute lymphocytic leukaemia	0.87 (0.59-1.29)	1.54 (0.97-2.45)
	Acute myeloid leukaemia	1.03 (0.86-1.24)	0.99 (0.88-1.10)
	Chronic lymphocytic leukaemia	1.75 (1.04-2.95)	1.72 (1.33-2.22)
	Chronic myeloid leukaemia	1.26 (0.65-2.44)	1.18 (0.76-1.82)
**All cancers combined**		1.22 (1.20-1.25)	1.01 (0.99-1.02)

[a] Excess mortality ratio comparing males to females, with follow-up up to five years after diagnosis. Adjusted for age and year of diagnosis.
[b] Corresponding to 95% Confidence intervals.

Table 6: Excess mortality ratios (EMRs) with 95% confidence intervals (CI) comparing males with females for 35 non-sex-specific cancer sites, by age groups 18-49 and 50-99, respectively

		**18-49 years**	**50-99 years**
**Cancer site**		**EMR[a] (95% CI)[b]**	**EMR[a] (95% CI)[b]**
**Head and neck**	Oral cavity	1.86 (1.27-2.73)	1.23 (1.11-1.36)
	Salivary glands	0.77 (0.29-2.06)	2.20 (1.56-3.11)
	Oropharynx	2.53 (0.99-6.45)	0.88 (0.73-1.06)
	Other pharynx	1.31 (0.88-1.94)	1.33 (1.17-1.51)
	Thyroid	3.23 (1.47-7.11)	1.76 (1.39-2.24)
**Digestive organs**	Oesophagus	0.93 (0.66-1.31)	1.04 (0.98-1.11)
	Stomach	1.00 (0.83-1.22)	1.03 (0.97-1.09)
	Small intestine	1.10 (0.60-2.04)	1.08 (0.92-1.25)
	Colon	1.03 (0.87-1.22)	1.03 (0.99-1.07)
	Rectal	1.10 (0.87-1.37)	1.09 (1.03-1.15)
	Anal	1.16 (0.63-2.12)	1.61 (1.31-1.99)
	Liver	0.99 (0.74-1.34)	1.05 (0.98-1.12)
	Gallbladder	0.60 (0.40-0.90)	0.93 (0.85-1.01)
	Pancreas	1.32 (1.09-1.60)	1.04 (1.00-1.08)
**Respiratory**	Nasal cavity	1.06 (0.50-2.25)	1.22 (0.95-1.57)
	Larynx	1.48 (0.74-2.99)	0.94 (0.80-1.11)
	Lung and trachea	1.28 (1.15-1.42)	1.17 (1.15-1.19)
	Pleura	0.65 (0.31-1.36)	1.18 (1.01-1.38)
**Bone and connective tissue**	Bone and cartilage	1.65 (0.99-2.75)	0.99 (0.66-1.50)
	Connective tissue	0.96 (0.73-1.28)	0.76 (0.66-0.87)
**Skin**	Malignant melanoma	2.03 (1.60-2.58)	1.82 (1.61-2.06)
	Non-melanoma	4.26 (1.46-12.49)	1.91 (1.60-2.28)
**Urinary tract**	Kidney	1.29 (0.95-1.75)	1.06 (0.98-1.14)
	Renal pelvis	0.82 (0.37-1.80)	0.90 (0.78-1.04)
	Bladder	0.44 (0.31-0.63)	0.73 (0.69-0.78)
**Central nervous system**	Eye	0.62 (0.25-1.51)	1.45 (1.05-2.00)
	Meninges	2.35 (0.95-5.79)	1.65 (1.29-2.09)
	Brain	1.47 (1.27-1.70)	1.23 (1.16-1.30)
**Hematological**	Hodgkin lymphoma	0.82 (0.38-1.76)	1.12 (0.82-1.52)
	Non-Hodgkin lymphoma	1.13 (0.84-1.52)	1.26 (1.17-1.35)
	Multiple myeloma	1.07 (0.66-1.73)	1.22 (1.11-1.34)
	Acute lymphocytic leukaemia	0.84 (0.49-1.44)	1.24 (0.86-1.77)
	Acute myeloid leukaemia	1.06 (0.76-1.47)	0.98 (0.89-1.09)
	Chronic lymphocytic leukaemia	3.54 (0.21-60.27)	1.71 (1.36-2.15)
	Chronic myeloid leukaemia	0.99 (0.34-2.89)	1.23 (0.83-1.80)
**All cancers combined**		1.45 (1.38-1.52)	1.06 (1.05-1.07)

[a] Excess mortality ratio comparing males to females, with follow-up up to five years after diagnosis. Adjusted for age and year of diagnosis.
[b] Corresponding to 95% Confidence intervals.

Table 7: Number (n), proportions (%), number of events, and person-years by cohabitation status, education level, and comorbidity (CCI) for 35 non-sex-specific cancer sites, for males and females included in the effect modification analyses

			Males			Females		
**Cancer site**	**Covariate**		**n (%)[a]**	**Events (%)[b]**	**Person-years[c]**	**n (%)[a]**	**Events (%)[b]**	**Person-years[c]**
**Oral cavity**	**Cohabitation status**	Living with a partner	1,962 (56)	700 (47)	6,301	989 (53)	298 (43)	3,298
		Living alone	1,513 (44)	785 (53)	3,919	878 (47)	388 (57)	2,359
	**Education level**	Short	1,229 (35)	577 (39)	3,560	876 (47)	373 (54)	2,532
		Medium	1,690 (49)	700 (47)	4,967	630 (34)	224 (33)	1,956
		Long	556 (16)	208 (14)	1,693	361 (19)	89 (13)	1,169
	**CCI**	0	2,546 (73)	977 (66)	7,893	1,394 (75)	432 (63)	4,536
		1	564 (16)	289 (19)	1,495	301 (16)	151 (22)	763
		2+	365 (11)	219 (15)	832	172 (9)	103 (15)	358
**Salivary glands**	**Cohabitation status**	Living with a partner	272 (70)	93 (62)	865	211 (63)	44 (56)	704
		Living alone	118 (30)	57 (38)	285	124 (37)	35 (44)	433
	**Education level**	Short	120 (31)	57 (38)	319	131 (39)	43 (54)	424
		Medium	179 (46)	72 (48)	523	123 (37)	21 (27)	428
		Long	91 (23)	21 (14)	308	81 (24)	15 (19)	285
	**CCI**	0	302 (77)	106 (71)	930	285 (85)	63 (80)	961
		1	54 (14)	25 (17)	150	32 (10)	8 (10)	119
		2+	34 (9)	19 (13)	70	18 (5)	8 (10)	56
**Oropharynx**	**Cohabitation status**	Living with a partner	649 (58)	183 (41)	2,082	180 (47)	61 (38)	572
		Living alone	473 (42)	261 (59)	1,127	200 (53)	101 (62)	499
	**Education level**	Short	388 (35)	185 (42)	966	153 (40)	82 (51)	397
		Medium	550 (49)	208 (47)	1,664	156 (41)	55 (34)	452
		Long	184 (16)	51 (11)	579	71 (19)	25 (15)	222
	**CCI**	0	831 (74)	284 (64)	2,543	284 (75)	113 (70)	838
		1	170 (15)	80 (18)	420	59 (16)	26 (16)	167
		2+	121 (11)	80 (18)	245	37 (10)	23 (14)	65
**Other pharynx**	**Cohabitation status**	Living with a partner	1,726 (58)	611 (45)	5,370	486 (54)	153 (45)	1,580

		Living alone	1,268 (42)	745 (55)	2,883	422 (46)	190 (55)	1,147
	**Education level**	Short	995 (33)	513 (38)	2,563	375 (41)	185 (54)	1,022
		Medium	1,461 (49)	665 (49)	3,966	347 (38)	110 (32)	1,091
		Long	538 (18)	178 (13)	1,723	186 (20)	48 (14)	613
	**CCI**	0	2,295 (77)	920 (68)	6,719	708 (78)	224 (65)	2,272
		1	383 (13)	221 (16)	944	115 (13)	64 (19)	262
		2+	316 (11)	215 (16)	589	85 (9)	55 (16)	192
**Thyroid**	**Cohabitation status**	Living with a partner	782 (75)	127 (73)	2,646	1,907 (72)	137 (51)	7,100
		Living alone	267 (25)	48 (27)	885	724 (28)	133 (49)	2,410
	**Education level**	Short	254 (24)	58 (33)	813	690 (26)	146 (54)	2,312
		Medium	469 (45)	78 (45)	1,601	1,029 (39)	75 (28)	3,832
		Long	326 (31)	39 (22)	1,117	912 (35)	49 (18)	3,367
	**CCI**	0	891 (85)	135 (77)	3,062	2,307 (88)	210 (78)	8,467
		1	106 (10)	26 (15)	325	222 (8)	33 (12)	716
		2+	52 (5)	14 (8)	144	102 (4)	27 (10)	327
**Oesophagus**	**Cohabitation status**	Living with a partner	3,005 (64)	2,362 (62)	4,342	802 (49)	604 (46)	1,185
		Living alone	1,722 (36)	1,464 (38)	1,826	833 (51)	696 (54)	895
	**Education level**	Short	1,721 (36)	1,436 (38)	2,098	841 (51)	707 (54)	945
		Medium	2,249 (48)	1,805 (47)	3,019	509 (31)	391 (30)	722
		Long	757 (16)	585 (15)	1,051	285 (17)	202 (16)	413
	**CCI**	0	3,370 (71)	2,690 (70)	4,610	1,188 (73)	912 (70)	1,657
		1	801 (17)	657 (17)	986	276 (17)	233 (18)	286
		2+	556 (12)	479 (13)	571	171 (10)	155 (12)	136
**Stomach**	**Cohabitation status**	Living with a partner	3,716 (72)	2,676 (70)	6,276	1,371 (55)	931 (52)	2,445
		Living alone	1,469 (28)	1,148 (30)	1,926	1,140 (45)	873 (48)	1,607
	**Education level**	Short	2,022 (39)	1,582 (41)	2,947	1,323 (53)	1,031 (57)	1,905
		Medium	2,307 (44)	1,646 (43)	3,805	772 (31)	514 (28)	1,347
		Long	856 (17)	596 (16)	1,449	416 (17)	259 (14)	799

	**CCI**	0	3,873 (75)	2,822 (74)	6,338	1,904 (76)	1,334 (74)	3,205
		1	826 (16)	624 (16)	1,205	409 (16)	311 (17)	607
		2+	486 (9)	378 (10)	659	198 (8)	159 (9)	239
**Small intestine**	**Cohabitation status**	Living with a partner	700 (72)	290 (68)	1,794	520 (61)	190 (53)	1,409
		Living alone	268 (28)	137 (32)	609	337 (39)	167 (47)	725
	**Education level**	Short	297 (31)	151 (35)	667	330 (39)	158 (44)	765
		Medium	443 (46)	180 (42)	1,165	308 (36)	126 (35)	745
		Long	228 (24)	96 (22)	571	219 (26)	73 (20)	623
	**CCI**	0	734 (76)	301 (70)	1,918	694 (81)	274 (77)	1,813
		1	141 (15)	71 (17)	307	105 (12)	52 (15)	208
		2+	93 (10)	55 (13)	178	58 (7)	31 (9)	113
**Colon**	**Cohabitation status**	Living with a partner	14,715 (72)	5,627 (66)	44,805	10,684 (54)	3,676 (45)	33,307
		Living alone	5,624 (28)	2,902 (34)	13,847	9,260 (46)	4,450 (55)	24,224
	**Education level**	Short	6,813 (33)	3,327 (39)	18,331	9,451 (47)	4,504 (55)	26,041
		Medium	9,124 (45)	3,639 (43)	26,864	6,857 (34)	2,474 (30)	20,419
		Long	4,402 (22)	1,563 (18)	13,457	3,636 (18)	1,148 (14)	11,071
	**CCI**	0	15,333 (75)	5,907 (69)	46,327	15,649 (78)	5,907 (73)	47,107
		1	3,024 (15)	1,449 (17)	7,938	2,835 (14)	1,361 (17)	7,262
		2+	1,982 (10)	1,173 (14)	4,387	1,460 (7)	858 (11)	3,163
**Rectal**	**Cohabitation status**	Living with a partner	9,261 (73)	3,218 (66)	30,913	4,506 (57)	1,294 (46)	15,556
		Living alone	3,429 (27)	1,672 (34)	9,219	3,405 (43)	1,522 (54)	9,854
	**Education level**	Short	4,325 (34)	1,971 (40)	12,960	3,620 (46)	1,559 (55)	11,116
		Medium	5,906 (47)	2,148 (44)	18,993	2,765 (35)	856 (30)	9,133
		Long	2,459 (19)	771 (16)	8,179	1,526 (19)	401 (14)	5,161
	**CCI**	0	10,028 (79)	3,558 (73)	32,939	6,652 (84)	2,171 (77)	22,055
		1	1,698 (13)	771 (16)	4,916	903 (11)	434 (15)	2,469
		2+	964 (8)	561 (11)	2,277	356 (5)	211 (7)	886
**Anal**	**Cohabitation status**	Living with a partner	303 (57)	96 (49)	1,002	652 (53)	143 (45)	2,302

		Living alone	231 (43)	98 (51)	668	578 (47)	174 (55)	1,879
	**Education level**	Short	195 (37)	83 (43)	581	451 (37)	157 (50)	1,442
		Medium	230 (43)	82 (42)	732	475 (39)	109 (34)	1,644
		Long	109 (20)	29 (15)	357	304 (25)	51 (16)	1,095
	**CCI**	0	400 (75)	137 (71)	1,296	1,000 (81)	220 (69)	3,519
		1	61 (11)	25 (13)	172	156 (13)	56 (18)	473
		2+	73 (14)	32 (16)	201	74 (6)	41 (13)	189
**Liver**	**Cohabitation status**	Living with a partner	2,200 (63)	1,915 (62)	2,114	701 (48)	583 (46)	754
		Living alone	1,317 (37)	1,157 (38)	1,167	770 (52)	676 (54)	676
	**Education level**	Short	1,309 (37)	1,165 (38)	1,125	758 (52)	683 (54)	667
		Medium	1,660 (47)	1,451 (47)	1,570	487 (33)	409 (32)	487
		Long	548 (16)	456 (15)	586	226 (15)	167 (13)	276
	**CCI**	0	1,735 (49)	1,524 (50)	1,594	894 (61)	758 (60)	905
		1	718 (20)	621 (20)	719	284 (19)	246 (20)	257
		2+	1,064 (30)	927 (30)	967	293 (20)	255 (20)	267
**Gallbladder**	**Cohabitation status**	Living with a partner	819 (71)	656 (69)	1,137	806 (54)	665 (53)	1,020
		Living alone	342 (29)	295 (31)	333	684 (46)	595 (47)	634
	**Education level**	Short	395 (34)	345 (36)	423	769 (52)	676 (54)	786
		Medium	515 (44)	419 (44)	671	474 (32)	397 (32)	588
		Long	251 (22)	187 (20)	375	247 (17)	187 (15)	280
	**CCI**	0	843 (73)	672 (71)	1,148	1,171 (79)	979 (78)	1,360
		1	180 (16)	155 (16)	208	219 (15)	190 (15)	216
		2+	138 (12)	124 (13)	113	100 (7)	91 (7)	77
**Pancreas**	**Cohabitation status**	Living with a partner	4,670 (71)	4,107 (70)	3,999	3,191 (52)	2,775 (51)	2,977
		Living alone	1,919 (29)	1,735 (30)	1,359	2,977 (48)	2,720 (49)	2,025
	**Education level**	Short	2,196 (33)	2,002 (34)	1,573	2,995 (49)	2,783 (51)	2,000
		Medium	3,016 (46)	2,653 (45)	2,577	2,048 (33)	1,776 (32)	1,856
		Long	1,377 (21)	1,187 (20)	1,208	1,125 (18)	936 (17)	1,146
	**CCI**	0	4,709 (71)	4,131 (71)	4,115	4,594 (74)	4,048 (74)	4,022

		1	1,089 (17)	978 (17)	789	1,032 (17)	949 (17)	710
		2+	791 (12)	733 (13)	454	542 (9)	498 (9)	271
**Nasal cavity**	**Cohabitation status**	Living with a partner	328 (65)	126 (58)	1,011	194 (56)	64 (48)	646
		Living alone	178 (35)	91 (42)	487	151 (44)	69 (52)	423
	**Education level**	Short	187 (37)	89 (41)	542	178 (52)	89 (67)	514
		Medium	244 (48)	104 (48)	730	102 (30)	26 (20)	359
		Long	75 (15)	24 (11)	227	65 (19)	18 (14)	197
	**CCI**	0	389 (77)	155 (71)	1,212	266 (77)	87 (65)	860
		1	72 (14)	39 (18)	189	58 (17)	32 (24)	159
		2+	45 (9)	23 (11)	98	21 (6)	14 (11)	51
**Larynx**	**Cohabitation status**	Living with a partner	1,654 (60)	524 (49)	5,896	296 (50)	96 (43)	997
		Living alone	1,105 (40)	549 (51)	3,054	295 (50)	125 (57)	920
	**Education level**	Short	1,146 (42)	487 (45)	3,553	304 (51)	130 (59)	956
		Medium	1,268 (46)	471 (44)	4,220	193 (33)	61 (28)	625
		Long	345 (13)	115 (11)	1,177	94 (16)	30 (14)	336
	**CCI**	0	1,998 (72)	695 (65)	6,844	426 (72)	137 (62)	1,460
		1	473 (17)	217 (20)	1,407	89 (15)	40 (18)	270
		2+	288 (10)	161 (15)	698	76 (13)	44 (20)	187
**Lung and trachea**	**Cohabitation status**	Living with a partner	19,663 (66)	15,910 (65)	25,331	14,624 (50)	10,652 (48)	23,304
		Living alone	10,063 (34)	8,572 (35)	10,370	14,382 (50)	11,465 (52)	18,252
	**Education level**	Short	12,728 (43)	10,818 (44)	14,208	16,138 (56)	12,914 (58)	21,506
		Medium	13,135 (44)	10,634 (43)	16,356	9,220 (32)	6,707 (30)	14,014
		Long	3,863 (13)	3,030 (12)	5,137	3,648 (13)	2,496 (11)	6,036
	**CCI**	0	19,804 (67)	16,181 (66)	25,039	20,316 (70)	15,209 (69)	30,769
		1	5,912 (20)	4,867 (20)	6,894	5,749 (20)	4,509 (20)	7,645
		2+	4,010 (13)	3,434 (14)	3,768	2,941 (10)	2,399 (11)	3,143
**Pleura**	**Cohabitation status**	Living with a partner	1,042 (76)	888 (76)	1,399	133 (53)	111 (52)	214
		Living alone	326 (24)	288 (24)	393	119 (47)	101 (48)	161

	**Education level**	Short	352 (26)	309 (26)	456	125 (50)	113 (53)	165
		Medium	737 (54)	637 (54)	938	84 (33)	62 (29)	133
		Long	279 (20)	230 (20)	397	43 (17)	37 (17)	77
	**CCI**	0	1,065 (78)	914 (78)	1,453	208 (83)	171 (81)	319
		1	206 (15)	178 (15)	233	32 (13)	30 (14)	46
		2+	97 (7)	84 (7)	106	12 (5)	11 (5)	10
**Bone and cartilage**	**Cohabitation status**	Living with a partner	250 (76)	79 (76)	830	155 (68)	32 (58)	538
		Living alone	79 (24)	25 (24)	263	72 (32)	23 (42)	197
	**Education level**	Short	84 (26)	31 (30)	282	59 (26)	25 (45)	156
		Medium	143 (43)	42 (40)	488	95 (42)	18 (33)	342
		Long	102 (31)	31 (30)	324	73 (32)	12 (22)	237
	**CCI[d]**	0	285 (87)	77 (74)	1,002	202 (89)	44 (80)	675
		1+	44 (13)	27 (26)	91	25 (11)	11 (20)	60
**Connective tissue**	**Cohabitation status**	Living with a partner	1,099 (75)	407 (72)	3,372	825 (61)	331 (56)	2,483
		Living alone	360 (25)	158 (28)	984	520 (39)	265 (44)	1,303
	**Education level**	Short	392 (27)	190 (34)	1,078	514 (38)	280 (47)	1,320
		Medium	697 (48)	254 (45)	2,131	459 (34)	195 (33)	1,281
		Long	370 (25)	121 (21)	1,147	372 (28)	121 (20)	1,185
	**CCI**	0	1,235 (85)	448 (79)	3,803	1,133 (84)	468 (79)	3,312
		1	145 (10)	75 (13)	354	144 (11)	80 (13)	355
		2+	79 (5)	42 (7)	199	68 (5)	48 (8)	119
**Malignant melanoma**	**Cohabitation status**	Living with a partner	10,424 (78)	1,458 (69)	38,439	10,307 (68)	643 (46)	40,371
		Living alone	2,970 (22)	660 (31)	10,028	4,910 (32)	746 (54)	18,044
	**Education level**	Short	2,863 (21)	717 (34)	9,982	3,770 (25)	686 (49)	14,230
		Medium	6,318 (47)	924 (44)	23,042	6,060 (40)	456 (33)	23,466
		Long	4,213 (31)	477 (23)	15,443	5,387 (35)	247 (18)	20,719
	**CCI**	0	11,316 (84)	1,497 (71)	42,063	13,537 (89)	998 (72)	52,713
		1	1,302 (10)	342 (16)	4,152	1,178 (8)	218 (16)	4,173
		2+	776 (6)	279 (13)	2,252	502 (3)	173 (12)	1,528

**Non-melanoma**	**Cohabitation status**	Living with a partner	12,368 (74)	2,460 (63)	42,047	6,260 (50)	575 (26)	22,217
		Living alone	4,437 (26)	1,457 (37)	13,794	6,330 (50)	1,618 (74)	20,177
	**Education level**	Short	5,580 (33)	1,669 (43)	18,482	5,724 (45)	1,423 (65)	19,147
		Medium	7,324 (44)	1,574 (40)	24,279	4,331 (34)	555 (25)	14,653
		Long	3,901 (23)	674 (17)	13,080	2,535 (20)	215 (10)	8,593
	**CCI**	0	11,389 (68)	1,860 (47)	40,187	9,306 (74)	1,132 (52)	32,785
		1	2,866 (17)	945 (24)	8,747	2,070 (16)	546 (25)	6,356
		2+	2,550 (15)	1,112 (28)	6,907	1,214 (10)	515 (23)	3,253
**Kidney**	**Cohabitation status**	Living with a partner	5,045 (72)	1,708 (67)	15,014	2,120 (59)	683 (51)	6,636
		Living alone	1,973 (28)	850 (33)	5,225	1,475 (41)	646 (49)	4,012
	**Education level**	Short	2,265 (32)	975 (38)	6,129	1,737 (48)	768 (58)	4,927
		Medium	3,269 (47)	1,139 (45)	9,507	1,243 (35)	392 (29)	3,846
		Long	1,484 (21)	444 (17)	4,602	615 (17)	169 (13)	1,875
	**CCI**	0	5,257 (75)	1,777 (69)	15,635	2,773 (77)	989 (74)	8,378
		1	1,005 (14)	430 (17)	2,764	481 (13)	194 (15)	1,363
		2+	756 (11)	351 (14)	1,839	341 (9)	146 (11)	907
**Renal pelvis**	**Cohabitation status**	Living with a partner	1,004 (74)	442 (71)	3,039	440 (49)	184 (42)	1,366
		Living alone	358 (26)	183 (29)	977	455 (51)	257 (58)	1,115
	**Education level**	Short	481 (35)	230 (37)	1,406	461 (52)	260 (59)	1,220
		Medium	616 (45)	285 (46)	1,834	304 (34)	128 (29)	884
		Long	265 (19)	110 (18)	777	130 (15)	53 (12)	378
	**CCI**	0	991 (73)	420 (67)	3,053	685 (77)	320 (73)	1,930
		1	204 (15)	103 (16)	558	137 (15)	75 (17)	382
		2+	167 (12)	102 (16)	405	73 (8)	46 (10)	169
**Bladder**	**Cohabitation status**	Living with a partner	12,198 (72)	3,928 (64)	43,788	2,908 (52)	910 (41)	10,376
		Living alone	4,644 (28)	2,172 (36)	14,211	2,672 (48)	1,324 (59)	7,654
	**Education level**	Short	6,078 (36)	2,601 (43)	19,976	2,901 (52)	1,391 (62)	8,643

		Medium	7,685 (46)	2,662 (44)	26,685	1,830 (33)	624 (28)	6,229
		Long	3,079 (18)	837 (14)	11,338	849 (15)	219 (10)	3,158
	**CCI**	0	12,178 (72)	3,821 (63)	43,797	4,221 (76)	1,505 (67)	14,391
		1	2,803 (17)	1,212 (20)	9,039	904 (16)	442 (20)	2,587
		2+	1,861 (11)	1,067 (17)	5,163	455 (8)	287 (13)	1,052
**Eye**	**Cohabitation status**	Living with a partner	427 (74)	101 (70)	1,544	357 (60)	63 (49)	1,422
		Living alone	152 (26)	43 (30)	530	234 (40)	66 (51)	824
	**Education level**	Short	156 (27)	47 (33)	546	212 (36)	58 (45)	808
		Medium	278 (48)	69 (48)	1,013	216 (37)	42 (33)	811
		Long	145 (25)	28 (19)	515	163 (28)	29 (22)	628
	**CCI**	0	479 (83)	99 (69)	1,785	483 (82)	96 (74)	1,871
		1	58 (10)	24 (17)	173	81 (14)	23 (18)	278
		2+	42 (7)	21 (15)	116	27 (5)	10 (8)	98
**Meninges**	**Cohabitation status**	Living with a partner	1,241 (71)	155 (57)	4,480	3,184 (62)	188 (35)	12,208
		Living alone	500 (29)	116 (43)	1,647	1,940 (38)	355 (65)	6,690
	**Education level**	Short	473 (27)	102 (38)	1,639	1,823 (36)	315 (58)	6,543
		Medium	796 (46)	123 (45)	2,789	1,902 (37)	153 (28)	7,213
		Long	472 (27)	46 (17)	1,699	1,399 (27)	75 (14)	5,142
	**CCI**	0	1,364 (78)	165 (61)	4,943	4,198 (82)	338 (62)	15,853
		1	251 (14)	55 (20)	839	645 (13)	111 (20)	2,231
		2+	126 (7)	51 (19)	345	281 (5)	94 (17)	813
**Brain and CNS**	**Cohabitation status**	Living with a partner	5,909 (75)	2,535 (74)	16,179	4,489 (65)	1,395 (57)	13,474
		Living alone	1,988 (25)	909 (26)	5,002	2,410 (35)	1,047 (43)	6,009
	**Education level**	Short	2,177 (28)	1,136 (33)	5,143	2,286 (33)	1,120 (46)	5,635
		Medium	3,614 (46)	1,549 (45)	9,984	2,538 (37)	813 (33)	7,441
		Long	2,106 (27)	759 (22)	6,054	2,075 (30)	509 (21)	6,409
	**CCI**	0	6,596 (84)	2,736 (79)	18,376	5,849 (85)	1,956 (80)	16,931
		1	866 (11)	452 (13)	1,970	735 (11)	313 (13)	1,904
		2+	435 (6)	256 (7)	834	315 (5)	173 (7)	649

**Hodgkin lymphoma**	**Cohabitation status**	Living with a partner	555 (69)	88 (63)	2,076	353 (67)	42 (45)	1,397
		Living alone	251 (31)	51 (37)	917	177 (33)	51 (55)	553
	**Education level**	Short	236 (29)	57 (41)	847	178 (34)	53 (57)	578
		Medium	372 (46)	56 (40)	1,398	200 (38)	28 (30)	765
		Long	198 (25)	26 (19)	748	152 (29)	12 (13)	606
	**CCI**	0	651 (81)	84 (60)	2,573	437 (82)	62 (67)	1,671
		1	92 (11)	30 (22)	264	69 (13)	18 (19)	216
		2+	63 (8)	25 (18)	156	24 (5)	13 (14)	63
**Non-Hodgkin lymphoma**	**Cohabitation status**	Living with a partner	6,460 (73)	1,817 (65)	21,287	3,927 (57)	784 (42)	13,784
		Living alone	2,390 (27)	958 (35)	6,761	2,968 (43)	1,084 (58)	8,791
	**Education level**	Short	2,807 (32)	1,095 (39)	8,356	2,996 (43)	1,081 (58)	9,356
		Medium	3,964 (45)	1,211 (44)	12,639	2,411 (35)	531 (28)	8,128
		Long	2,079 (23)	469 (17)	7,054	1,488 (22)	256 (14)	5,091
	**CCI**	0	6,876 (78)	1,871 (67)	22,783	5,414 (79)	1,294 (69)	18,423
		1	1,236 (14)	520 (19)	3,493	1,043 (15)	363 (19)	3,087
		2+	738 (8)	384 (14)	1,772	438 (6)	211 (11)	1,065
**Multiple myeloma**	**Cohabitation status**	Living with a partner	2,501 (76)	1,050 (73)	7,282	1,420 (58)	469 (47)	4,477
		Living alone	772 (24)	398 (27)	1,841	1,041 (42)	532 (53)	2,687
	**Education level**	Short	1,009 (31)	518 (36)	2,635	1,063 (43)	549 (55)	2,976
		Medium	1,516 (46)	620 (43)	4,310	810 (33)	283 (28)	2,395
		Long	748 (23)	310 (21)	2,178	588 (24)	169 (17)	1,794
	**CCI**	0	2,500 (76)	1,029 (71)	7,303	1,984 (81)	738 (74)	5,990
		1	460 (14)	238 (16)	1,153	323 (13)	166 (17)	833
		2+	313 (10)	181 (12)	667	154 (6)	97 (10)	341
**Acute lymphocytic leukaemia**	**Cohabitation status**	Living with a partner	126 (78)	68 (77)	302	84 (64)	41 (56)	234
		Living alone	35 (22)	20 (23)	84	48 (36)	32 (44)	88
	**Education level**	Short	52 (32)	40 (45)	96	43 (33)	33 (45)	77
		Medium	79 (49)	33 (38)	214	55 (42)	26 (36)	139
		Long	30 (19)	15 (17)	76	34 (26)	14 (19)	106

	**CCI**[d]	0	128 (80)	68 (77)	322	118 (89)	63 (86)	300
		1+	33 (20)	20 (23)	64	14 (11)	10 (14)	22
**Acute myeloid leukaemia**	**Cohabitation status**	Living with a partner	952 (70)	683 (68)	1,641	576 (54)	368 (48)	1,170
		Living alone	402 (30)	321 (32)	537	494 (46)	396 (52)	607
	**Education level**	Short	464 (34)	382 (38)	601	461 (43)	377 (49)	586
		Medium	632 (47)	450 (45)	1,093	382 (36)	270 (35)	664
		Long	258 (19)	172 (17)	483	227 (21)	117 (15)	527
	**CCI**	0	1,050 (78)	741 (74)	1,882	864 (81)	591 (77)	1,544
		1	186 (14)	155 (15)	204	149 (14)	124 (16)	187
		2+	118 (9)	108 (11)	91	57 (5)	49 (6)	47
**Chronic lymphocytic leukaemia**	**Cohabitation status**	Living with a partner	2,446 (76)	452 (65)	9,365	1,114 (56)	104 (30)	4,624
		Living alone	781 (24)	242 (35)	2,689	870 (44)	237 (70)	3,192
	**Education level**	Short	1,048 (32)	303 (44)	3,861	893 (45)	225 (66)	3,465
		Medium	1,417 (44)	288 (41)	5,282	661 (33)	85 (25)	2,601
		Long	762 (24)	103 (15)	2,910	430 (22)	31 (9)	1,750
	**CCI**	0	2,585 (80)	471 (68)	9,964	1,674 (84)	259 (76)	6,682
		1	402 (12)	120 (17)	1,329	220 (11)	48 (14)	829
		2+	240 (7)	103 (15)	760	90 (5)	34 (10)	306
**Chronic myeloid leukaemia**	**Cohabitation status**	Living with a partner	319 (73)	58 (62)	1,163	223 (65)	31 (44)	822
		Living alone	120 (27)	36 (38)	393	120 (35)	39 (56)	379
	**Education level**	Short	125 (28)	38 (40)	404	118 (34)	44 (63)	355
		Medium	211 (48)	43 (46)	766	136 (40)	18 (26)	490
		Long	103 (23)	13 (14)	386	89 (26)	8 (11)	355
	**CCI**	0	353 (80)	67 (71)	1,284	293 (85)	52 (74)	1,059
		1	55 (13)	15 (16)	169	32 (9)	10 (14)	97
		2+	31 (7)	12 (13)	104	18 (5)	8 (11)	45
**All cancers combined**	**Cohabitation status**	Living with a partner	130,533 (71)	57,452 (65)	358,504	80,899 (56)	29,275 (47)	236,723
		Living alone	53,708 (29)	30,513 (35)	119,968	63,909 (44)	33,132 (53)	151,239
	**Education level**	Short	62,784 (34)	35,174 (40)	148,762	64,643 (45)	34,952 (56)	155,303

		Medium	83,962 (46)	38,827 (44)	221,451	50,143 (35)	18,973 (30)	141,016
		Long	37,495 (20)	13,964 (16)	108,259	30,022 (21)	8,482 (14)	91,643
	**CCI**	0	136,107 (74)	59,190 (67)	376,095	112,958 (78)	44,080 (71)	320,270
		1	28,377 (15)	15,980 (18)	64,460	21,086 (15)	11,450 (18)	47,508
		2+	197,57 (11)	12,795 (15)	37,917	10,764 (7)	6,877 (11)	20,184

[a] Number and proportion (%) of cancer cases in the cohort. Study population restricted to individuals diagnosed with cancer in 2004-2020, aged ≥ 30 years at diagnosis, born after 1920, not immigrated < 5 years prior to diagnosis, and no missing information on cohabitation status and education.

[b] Number and proportion (%) of deaths up to five years from date of diagnosis.

[c] Number of person-years. Individuals were followed from date of diagnosis until death, emigration or last date of follow-up (Five years from diagnosis or December 31, 2020), whichever occurred first.

[d] CCI groups ≥ 1 collapsed due to few observations in some groups.

STROBE Statement—checklist of items that should be included in reports of observational studies

	Item No.	Recommendation	Page No.	Relevant text from manuscript
**Title and abstract**	1	(*a*) Indicate the study's design with a commonly used term in the title or the abstract	1	***Title***
		(*b*) Provide in the abstract an informative and balanced summary of what was done and what was found	2	***Methods and Results***
**Introduction**				
Background/rationale	2	Explain the scientific background and rationale for the investigation being reported	4	***Introduction***
Objectives	3	State specific objectives, including any prespecified hypotheses	4	***Introduction***
**Methods**				
Study design	4	Present key elements of study design early in the paper	5	***Study population and data sources***
Setting	5	Describe the setting, locations, and relevant dates, including periods of recruitment, exposure, follow-up, and data collection	5-7	***Study population and data sources, statistical analyses***
Participants	6	(*a*) *Cohort study*—Give the eligibility criteria, and the sources and methods of selection of participants. Describe methods of follow-up	5-7, Supplementary Figure 1	***Study population and data sources, statistical analyses***
		(*b*) *Cohort study*—For matched studies, give matching criteria and number of exposed and unexposed	Not relevant	
Variables	7	Clearly define all outcomes, exposures, predictors, potential confounders, and effect modifiers. Give diagnostic criteria, if applicable	5-7	***Study population and data sources, statistical analyses***
Data sources/ measurement	8*	For each variable of interest, give sources of data and details of methods of assessment (measurement). Describe comparability of assessment methods if there is more than one group	5-7	***Study population and data sources, statistical analyses***
Bias	9	Describe any efforts to address potential sources of bias	6-7	***Statistical analyses***

Study size	10	Explain how the study size was arrived at	5-6, Supplementary Figure 1, Supplementary Table 3	***Study population and data sources***
Quantitative variables	11	Explain how quantitative variables were handled in the analyses. If applicable, describe which groupings were chosen and why	5-7	***Study population and data sources, statistical analyses***
Statistical methods	12	(*a*) Describe all statistical methods, including those used to control for confounding	6-7	***Statistical analyses***
		(*b*) Describe any methods used to examine subgroups and interactions	7	***Statistical analyses***
		(*c*) Explain how missing data were addressed	5-6, Supplementary Table 3	***Study population and data sources***
		(*d*) *Cohort study*—If applicable, explain how loss to follow-up was addressed	6	***Statistical analyses***
		(*e*) Describe any sensitivity analyses	7	***Statistical analyses***
**Results**				
Participants	13*	(a) Report numbers of individuals at each stage of study—eg numbers potentially eligible, examined for eligibility, confirmed eligible, included in the study, completing follow-up, and analysed	7, Supplementary Figure 1, Supplementary Table 3	***Results***
		(b) Give reasons for non-participation at each stage	Supplementary Figure 1	
		(c) Consider use of a flow diagram	Supplementary Figure 1	
Descriptive data	14*	(a) Give characteristics of study participants (eg demographic, clinical, social) and information on exposures and potential confounders	7, Table 1, Supplementary Table 4,	***Results***

			Supplementary Table 7	
		(b) Indicate number of participants with missing data for each variable of interest	Supplementary Table 3	
		(c) *Cohort study*—Summarize follow-up time (eg, average and total amount)	7, Table 1, Supplementary Table 4, Supplementary Table 7	***Results***
Outcome data	15*	*Cohort study*—Report numbers of outcome events or summary measures over time	Table 1, Supplementary Table 4, Supplementary Table 7	
Main results	16	(*a*) Give unadjusted estimates and, if applicable, confounder-adjusted estimates and their precision (eg, 95% confidence interval). Make clear which confounders were adjusted for and why they were included	8-9, Table 2, Supplementary Figure 2, Supplementary Table 5, Supplementary Table 6	***Results***
		(*b*) Report category boundaries when continuous variables were categorized	7, Supplementary Table 5, Supplementary Table 6	***Statistical analyses, Results***

		(*c*) If relevant, consider translating estimates of relative risk into absolute risk for a meaningful time period	8, Table 2	***Results***
Other analyses	17	Report other analyses done—eg analyses of subgroups and interactions, and sensitivity analyses	8-9, Supplementary Figure 2, Supplementary Table 5 Supplementary Table 6	***Results***
**Discussion**				
Key results	18	Summarize key results with reference to study objectives	10-11	***Discussion***
Limitations	19	Discuss limitations of the study, taking into account sources of potential bias or imprecision. Discuss both direction and magnitude of any potential bias	11-12	***Discussion***
Interpretation	20	Give a cautious overall interpretation of results considering objectives, limitations, multiplicity of analyses, results from similar studies, and other relevant evidence	10	***Discussion***
Generalizability	21	Discuss the generalizability (external validity) of the study results	12	***Discussion***
**Other information**				
Funding	22	Give the source of funding and the role of the funders for the present study and, if applicable, for the original study on which the present article is based	14	***Funding information***
